# Overexpression of *TgERF1*, a Transcription Factor from *Tectona grandis*, Increases Tolerance to Drought and Salt Stress in Tobacco

**DOI:** 10.3390/ijms24044149

**Published:** 2023-02-19

**Authors:** Perla Novais de Oliveira, Fernando Matias, Cristina Martínez-Andújar, Purificación Andrea Martinez-Melgarejo, Ángela Sánchez Prudencio, Esteban Galeano, Francisco Pérez-Alfocea, Helaine Carrer

**Affiliations:** 1 Department of Biological Sciences, Luiz de Queiroz College of Agriculture (ESALQ), University of São Paulo, Piracicaba 13418-900, Brazil; 2 Department of Plant Nutrition, Centro de Edafología Aplicada del Segura (CEBAS)-CSIC, 30100 Murcia, Spain; 3 Department of Forestry, Mississippi State University, Starkville, MS 39762, USA

**Keywords:** *Tectona grandis*, tropical tree, AP2/ERF family, drought stress, salt stress

## Abstract

Teak (*Tectona grandis*) is one of the most important wood sources, and it is cultivated in tropical regions with a significant market around the world. Abiotic stresses are an increasingly common and worrying environmental phenomenon because it causes production losses in both agriculture and forestry. Plants adapt to these stress conditions by activation or repression of specific genes, and they synthesize numerous stress proteins to maintain their cellular function. For example, APETALA2/ethylene response factor (AP2/ERF) was found to be involved in stress signal transduction. A search in the teak transcriptome database identified an *AP2/ERF* gene named *TgERF1* with a key AP2/ERF domain. We then verified that the *TgERF1* expression is rapidly induced by Polyethylene Glycol (PEG), NaCl, and exogenous phytohormone treatments, suggesting a potential role in drought and salt stress tolerance in teak. The full-length coding sequence of *TgERF1* gene was isolated from teak young stems, characterized, cloned, and constitutively overexpressed in tobacco plants. In transgenic tobacco plants, the overexpressed TgERF1 protein was localized exclusively in the cell nucleus, as expected for a transcription factor. Furthermore, functional characterization of *TgERF1* provided evidence that *TgERF1* is a promising candidate gene to be used as selective marker on plant breeding intending to improve plant stress tolerance.

## 1. Introduction

Abiotic stresses, such as high salinity, drought, cold, and alkalinity, are the most common factors restricting plant growth, development and crop yields [1]. Plants adapt to these stress conditions by altering physiology, morphology, and levels of molecular compounds [2]. Under environmental stress, plants activate multiple defense mechanisms through the activation or repression of specific genes leading to the synthesis of numerous stress proteins to maintain cellular function in stress response and plant defense [3,4]. Transcription factors (TFs) play important roles in the regulation of these responsive genes in plants [4], through binding to *cis*-acting elements in the promoters of downstream target genes or by interacting with other transcription factors [5]. For example, *WRKY*, *NAC* (NAM, ATAF1, 2, and CUC2), *HLH* (base helix-loop-helix transcription factors), *MYB* (myeloblastosis oncogene), *bZIP* (basic leucine zipper), and *AP2/ERF* genes were found to play important roles in stress signal transduction [6,7,8,9].

AP2/ERF TFs belong to one of the largest plant kingdom families of transcription factors and they are characterized by the AP2/ERF domain, mainly composed of approximately 60 amino acids [10]. These amino acids bind to various dehydration-responsive elements/C-repeats (DRE/CRT) and the GCC box [11]. AP2/ERF TFs have been shown to play essential roles in multiple biological processes that regulate plant growth and development [12], as well as responses to environmental stresses [10,13]. Previous studies have shown that the expression levels of these genes can be induced by environmental signals such as drought, salt, cold and other stresses, which are often mediated by hormonal signaling [1,5,14,15,16]. The AP2/ERF superfamily has been classified into groups based on their sequence similarities and numbers of AP2/ERF domains: AP2, CRT/DRE binding factor (CBF/DREB), ethylene-responsive factor (ERF), and related to ABI3/VP1 (RAV) [10]. ERF proteins were first reported in tobacco [17], and since then, several ERF proteins have been identified in a series of plant species, such as Arabidopsis, tomato, wheat, rice, poplar, eucalyptus, and apple [10,18,19,20,21,22,23]. The ERFs’ proteins contain a highly conserved DNA-binding domain (ERF domain) that interacts with the GCC-box; this plays important roles in responses to biotic stress [24]. However, the ERF protein also binds to DRE/CRT, mainly involved in responses to drought, high salt, and extreme temperatures [25,26]. These proteins have been studied extensively during the past few years. For instance, overexpression of the *Jatropha curcas JcERF* gene in Arabidopsis increased salt tolerance [27]. The overexpression of *TaERF3*, an AP2/ERF type transcription factor, significantly increased salt and drought stress tolerance of transgenic wheat [28]. It has been reported that overexpression of the *GmERF3* soybean gene in tobacco can enhance salt and drought stress tolerance [29].

*Tectona grandis* Linn. f., popularly known as teak (Lamiaceae family), is one of the most important wood sources due to its extraordinary qualities of color, density, and durability, and it is cultivated in tropical regions with a noteworthy market around the world [30]. Abiotic stress is an increasingly common and worrying phenomenon because it causes a loss of production in agriculture and forestry. Teak is a tropical tree that requires alternating rainy and dry seasons to produce high-quality wood. Thus, there is a concern for its cultivation in areas with a water deficit, given the relatively high transpiration rates of this species that result in reduced diameter growth [31].

We identified an *AP2/ERF* gene that contains a key AP2/ERF domain in the teak transcriptome database, named *TgERF1* (GenBank accession No. MH003850.1). Based on the discovered functions of AP2/ERF-related genes from diverse plants, and on the potential role of *TgERF1* in regulating abiotic stress tolerance, the following objectives were proposed for this study: (1) to isolate, characterize and clone the *TgERF1* gene and constitutively overexpress it in tobacco plants; (2) to investigate the role of *TgERF1* in the regulation of responses to drought and salt stress in tobacco transgenic plants; (3) to investigate the subcellular localization of the *TgERF1* gene in tobacco transgenic plants; and (4) to study the response of *TgERF1* by PEG, Mannitol, and NaCl treatments in tobacco transgenic plants. To our knowledge, this is the first study cloning an abiotic stress-related gene from teak followed by transformation and overexpression in a model plant species, which was then exposed to drought and salinity stress.

## 2. Results

### 2.1. Identification and Phylogenetic Analyses of AP2/ERF Transcription Factors in Teak

**Figure 1:**
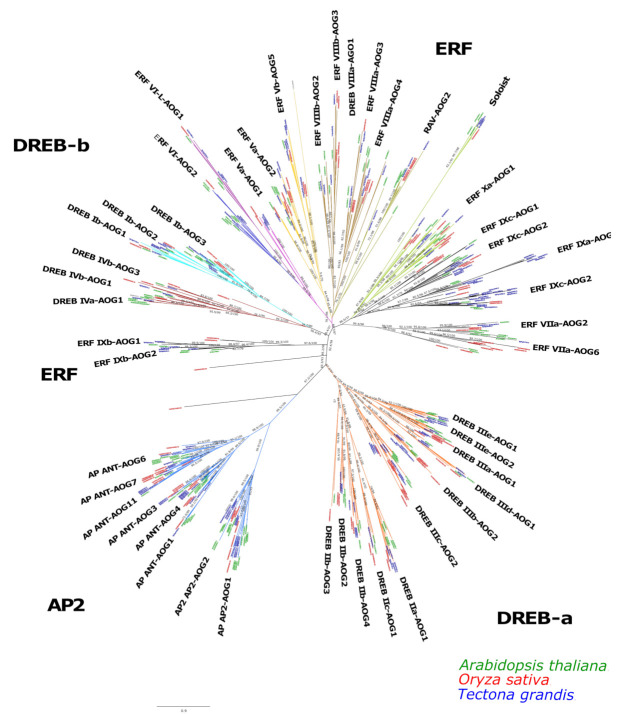
Phylogenetic tree for the AP2/ERF family transcription factors of teak, Arabidopsis, and rice. The transcription factors of teak are shown in blue, Arabidopsis in green, and rice in red. A consensus tree is shown. Subgroups’ names were classified according to Wang (2019) [32]. The scale bar indicates the substitution rate per residue.

A total of 201 nonredundant AP2/ERF transcription factors (100%) were identified in teak. The same pipeline applied to Arabidopsis identified 184 proteins and 135 for rice. In the phylogenetic tree, the AP2/ERF proteins were classified into three subfamilies according to their number of AP2/ERF domains and branch topology: AP2, DREB, and ERF. Each sub-family was classified into groups, which were named according to [32]. Figure 1 depicts the identified groups in each subfamily, which totalized 8 in the AP2, 17 in the DREB, and 21 in the ERF. Three orphan sequences were found, and notably, these sequences belong to rice. In general, teak AP2 proteins were distributed in at least one group (Figure 1).

### 2.2. Phylogenetic Tree and Structure of AP2/ERF Transcription Factors in Teak

**Figure 2:**
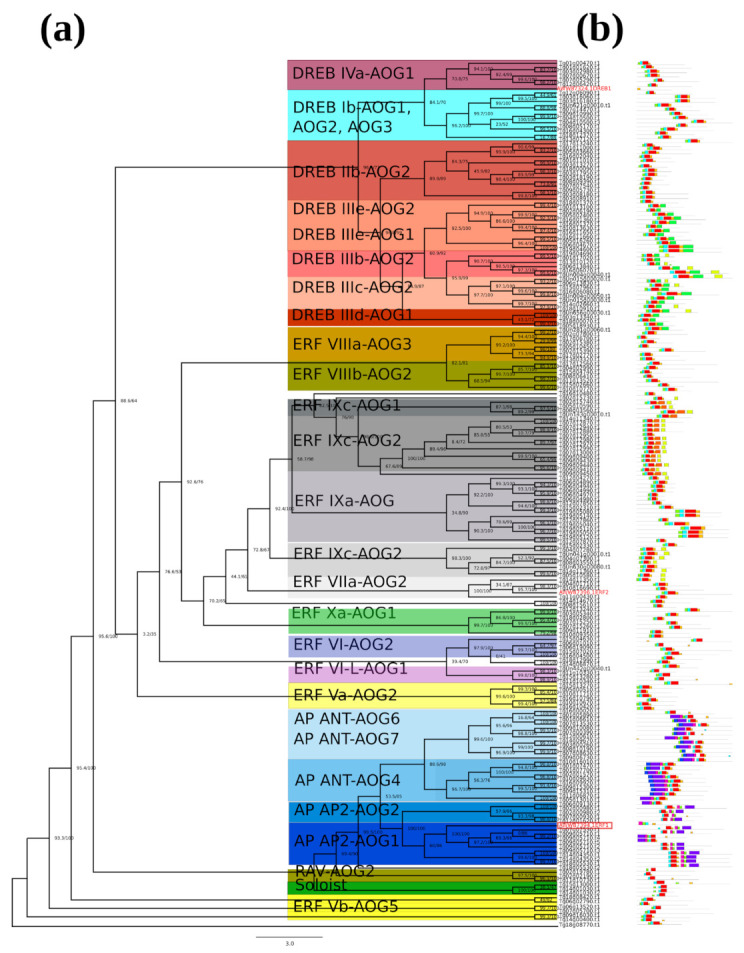
Phylogenetic tree and conserved protein motif for the teak AP2/ERF family of transcription factors. (**a**) The AWW87324, AWW87394, and AWW87396 proteins were aligned for construction of the phylogenetic tree. The consensus tree (after 1000 bootstrap samplings) with teak subgroup names is shown. The branch of AP2/ERF classification for teak is shown in blue, Arabidopsis in green, and rice in red. The scale bar indicates the substitution rate per residue. The motif composition of teak AP2/ERF proteins and the ten motifs are displayed in different colored boxes (**b**).

Evolutionary relationships of teak AP2/ERF transcription factors were analyzed in another unrooted phylogenetic tree based on the amino acid sequence of 201 teak proteins. Results were complemented with motif structure detection (Figure 2a,b). Indeed, 26 groups were identified among AP2/ERF teak proteins. The motif structure pattern was similar in ERF and DREB subfamilies, and the AP2 subfamily especially showed two highly conserved motifs represented in blue and purple boxes (Figure 2b).

**Figure 3:**
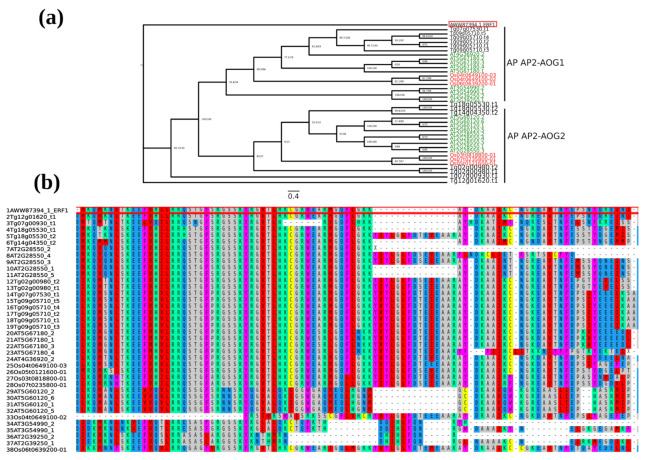
Multiple sequence alignment and phylogenetic tree of AP AP2-AOG1 and -AOG2 subgroups of AP2/ERF family transcription factors. (**a**) AP AP2-AOG1 and -AOG2 subgroups including AWW87394 (TgERF1) orthologous protein is shown in black, Arabidopsis in green, and rice in red, (**b**) TgERF1 multiple alignment to AP AP2-AOG1 and -AOG2 subgroups orthologous protein.

The AWW87324.1 (DREB1) protein is located in the DREB IVa-AOG1 and AWW87394.1 (ERF1) in AP AP2-AOG2. Analysis of evolutionary relationships between the rice TgERF1 protein and the Arabidopsis AP2 protein revealed six TgERF1 paralogous and nine orthologous in Arabidopsis (Figure 3a). The proximal orthologue is the AT2G28550 splice variant 2, annotated as related to AP2.7 (RAP2.7) or TOE1 in the Araport11. These results were confirmed by multiple sequence alignment, showing that AT2G28550.2 presents the highest similarity sequence to TgERF1 (Figure 3b).

### 2.3. Expression Pattern of the TgERF1 Gene in Response to Abiotic Stress and Exogenous Phytohormone Treatments in Teak Plants

**Figure 4:**
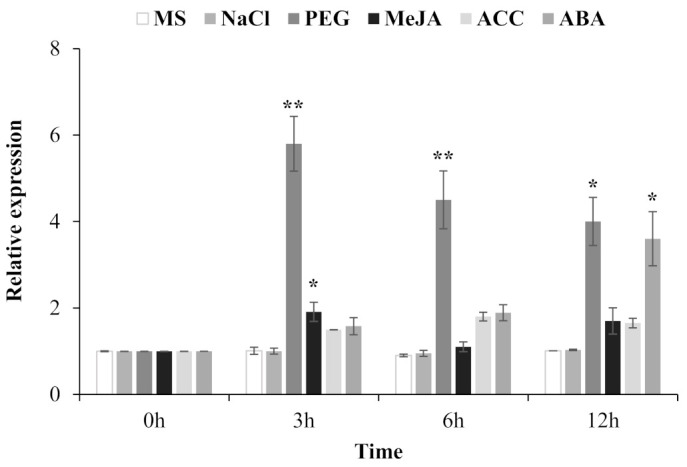
Expression profiles of *TgEREF1* in response to ionic saline (NaCl) and non-ionic osmotic (PEG) stresses, and phytohormones in teak (*Tectona grandis*), as detected by RT-qPCR. Total RNA was isolated from leaves of two-month-old teak seedlings that were sampled after exposure to 150 mM NaCl, 20% PEG, 100 μM MeJA, 100 μM ACC, and 100 μM ABA. Controls consisted of teak seedlings incubated on MS culture medium. Values correspond to the means ± standard error of the mean of three biological replicates. * *p* < 0.05 and ** *p* < 0.01.

At first, expression patterns of *TgERF1* in stems, leaves, and roots were characterized in RT-PCR analyses. This gene was expressed in all the studied organs (Figure S1 (Supplementary Materials)) with the highest level of expression in leaves and roots. ERFs are known to play a direct regulatory role in response to abiotic stresses and phytohormonal stimuli [13]. Thus, the *TgERF1* expression was analyzed by RT-qPCR in shoots of 60-day-old teak plants exposed on diverse levels of NaCl and PEG, and also under the hormones such as methyl jasmonate (MeJA), ethylene-precursor 1-aminocyclopropane-1-carboxylic acid (ACC), and abscisic acid (ABA) assessed within a 12-h interval (Figure 4). The *TgERF1* gene did not respond to NaCl treatments, probably because the plants were growing under in vitro conditions and with low transpiration rates. It is possible that they would then require an additional time period to be sensitive to salinity. However, PEG stress induced high levels of transcript accumulation, which reached their maximum at 3 h of treatment and decreased thereafter showing a significantly high sensitivity to osmotic pressure. As for phytohormones, MeJA induced significant accumulation levels of *TgERF1* mRNA up to 3 h treatment, which then decreased at 6 h, and subsequently increased at 12 h. Under the ACC treatment, *TgERF1* mRNA levels accumulated at 3 h treatment, reached a maximum level at 6 h, and up to 12 h they decreased to levels similar to the 3 h treatment. The ABA phytohormone induced progressive expression of *TgERF1* transcripts, which reached their maximum at 12 h of treatment. In conjunction, these expression data indicated that *TgERF1* may be involved in the plant response to various stresses through different signaling mechanisms.

### 2.4. Subcellular Localization of TgERF1 Protein

**Figure 5:**
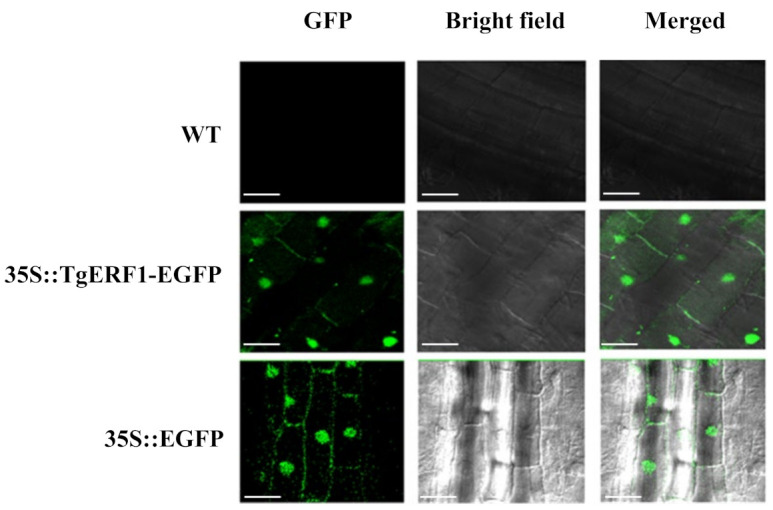
Subcellular localization of TgERF1::GFP protein in roots of transgenic tobacco seedlings. Cells from the root elongation zone of 7-day-old seedlings of a tobacco line were analyzed under Fluorescence Excitation (GFP), Bright Field, and Merge contrasting in a confocal microscope. WT root tissues without fluorescence; Transgenic line 35S::TgERF1- EGFP showing GFP fluorescence in the nucleus; Control line 35S::EGFP showing GFP fluorescence in the nucleus and cytoplasm. Bar: 30 μm.

To investigate the subcellular localization of the TgERF1 protein, tobacco leaves were transformed with the empty and 35S::TgERF1-EGFP vectors via *Agrobacterium tumefasciens*. As expected, the wild-type (WT) tobacco plants showed no fluorescence (Figure 5). The GFP fluorescence from the TgERF1-EGFP protein was observed in the nucleus with greater intensity, which was confirmed by merge contrasting (Figure 5). This result is in accordance with the in silico analysis, which predicted that the TgERF1 protein is localized in the nucleus (Table S1) since this the protein lacks the transmembrane motif (Table S2). Tobacco plants transformed with the 35S::GFP control vector showed GFP signals in the entire cytoplasm and nucleus (Figure 5). These results demonstrated that TgERF1 is a nuclear-localized protein.

### 2.5. Identification of TgERF1 Transgenic Tobacco Plants

**Figure 6:**
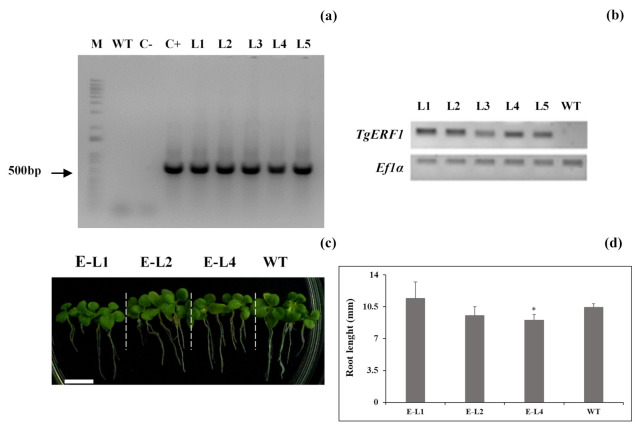
Molecular and phenotypic characterization of transgenic tobacco plants overexpressing the *TgERF1* gene. (**a**) Representative electrophoresis analysis of PCR amplification of the 35S::TgERF1 (520 bp fragment). Lane M: molecular weight marker (1-kb DNA ladder); Lane WT: wild-type genomic DNA. Lane C-: reaction mixture without DNA template as a negative control. Lane C+: plasmid DNA as a positive control. Lane L1, L2, L3, L4, and L5: kanamycin-resistant plants from putative transgenic events; (**b**) Expression of *TgERF1* in WT and transgenic lines. Leaf tissues were examined by semiquantitative RT-PCR. The constitutive *NtEF1α* (*Elongation Factor-1 alpha*) housekeeping gene was used as an internal control; (**c**) Picture of 7-day-old WT and transgenic seedlings (E-L1, E-L2, E-L4) grown on MS culture medium. Bar: 1 cm; (**d**) Roots’ elongation rates. Bars represent a mean value of 20 seedlings/line ± SEM. Significant differences were determined by ANOVA followed by Dunnett’s test. * Significant at *p* < 0.05.

A total of 16 independent transgenic lines (T0) were selected by kanamycin-resistance screening, and five homozygous transgenic lines overexpressing *TgERF1* were developed and confirmed by PCR analysis using cauliflower mosaic virus (CaMV) 35S forward and gene-specific reverse primers (Table S5). As shown in Figure 6a, a specific 503-bp band was PCR amplified in the L1 to L5 transgenic tobacco lines. Subsequently, *TgERF1* expression levels were analyzed by semiquantitative RT-PCR (Figure 6b). Of the five selected transgenic lines, three (L1, L2, and L4) expressed high levels of *TgERF1* transcripts while they were relatively low in the other two lines (L3 and L5). Moreover, no *TgERF1* expression was detected in WT plants. Therefore, L1, L2, and L4 were selected for further experiments. The PCR results demonstrated that the *TgERF1* gene was successfully introduced and expressed in tobacco plants. For the seedlings grown in MS culture medium, no significant differences in root length were observed in 7-day-old L1 and L2 plants compared to WT plants, but roots were shorter in L4 plants (Figure 6c,d).

### 2.6. Role of TgERF1 during Post-Germinative Root Development under Abiotic Stresses

**Figure 7:**
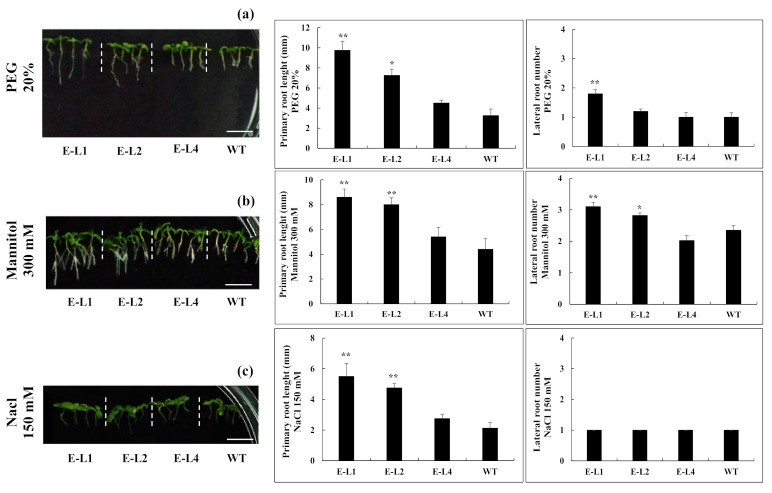
Root growth of transgenic tobacco lines overexpressing the *TgERF1* gene and WT seedlings under various stress treatments, which consisted of (**a**) 20% PEG, (**b**) 300 mM mannitol, and (**c**) 150 mM NaCl. Bar: 1 cm. In these experiments, three-day-old seedlings were transferred to the osmotic and saline treatments and grown for seven days. The primary root length and lateral root numbers were recorded at seven days after the transfer. Each experiment consisted of 16 seedlings/line and three repetitions. Bars represent the mean value ± SEM of three independent assays. Significant differences were determined by ANOVA followed by Dunnett’s test. ** Significant at *p* < 0.01 and * at *p* < 0.05.

In order to investigate the possible effects on stress tolerance of the constitutive expression of *TgERF1* in transgenic plants, three-day-old 35S::ERF1 and WT seedlings were grown for ten days on MS culture media supplemented with either PEG (0, 5, 10, and 20% W/V), mannitol (0, 100, 200, and 300 mM), or NaCl (0, 50, 150, and 200 mM). As shown in Figure 7, under all PEG concentrations there were significant differences in root length between the L1 transgenic line overexpressing *TgERF1* and the WT control (Figure S2). The root length of the L1 line in the presence of 20% PEG was 2.7-fold greater compared to WT seedlings. There were no significant root length differences comparing L2 and WT seedlings under 0, 5, or 10% PEG stress conditions (Figure S2). However, the root growth of L2 seedlings under 20% PEG stress was increased by 38.5% compared to WT seedlings. Considering all PEG stress conditions, there were no significant root length differences between the L4 line and the WT control (Figure S2). As for lateral root numbers, L1 was the only line that differed significantly from the WT control under the various PEG concentrations (Figure S2).

Seedlings of transgenic lines overexpressing *TgERF1* and of the WT control were grown for under mannitol treatments (Figure S3). As illustrated in Figure 7b, L1-L2 transgenic lines showed similar root growth responses to 20% PEG and 300 mM mannitol. They exhibited longer primary root length and higher numbers of lateral roots compared to WT seedlings. There were no significant differences in the primary root length of L2-L4 lines compared to WT seedlings (Figure S3) treated with 100 or 200 mM mannitol. Furthermore, there were no significant differences for numbers of lateral roots (Figure S3).

As for salt stress tolerance, significant phenotypic differences were observed among WT, L1, and L2 lines at seven days of stress in all NaCl concentrations (Figure S4). Seedlings of L1 and L2 lines exhibited longer primary roots than the WT control. On the contrary, root growth in the L4 line did differ significantly from WT seedlings. As shown in Figure S4, transgenic lines exhibited similar lateral root numbers compared to WT seedlings. In conjunction, these results indicated that the *TgERF1* overexpression confers tolerance to osmotic/water/saline abiotic stress in transgenic plants during the post-germinative growth stage by improving the growth of the primary roots.

### 2.7. Role of TgERF1 under Salt Stress

**Figure 8:**
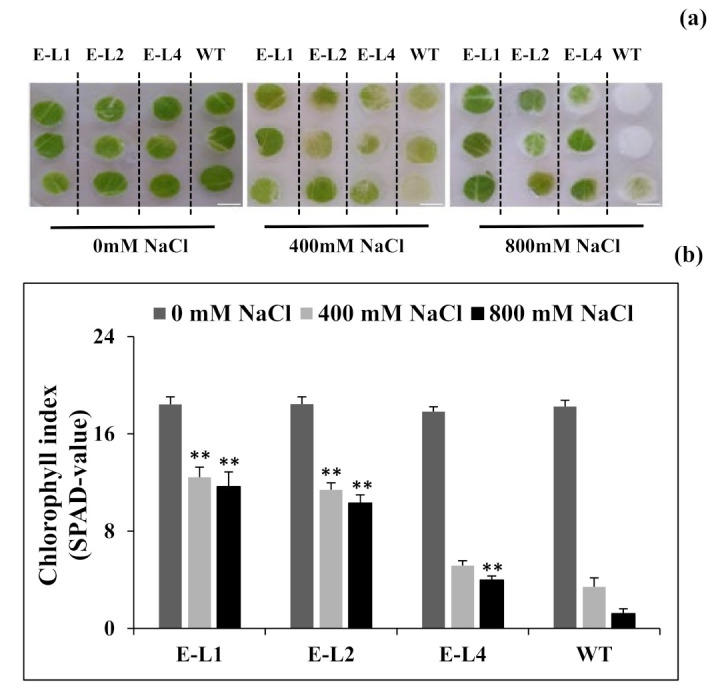
Representative phenotypes in transgenic tobacco lines and WT leaf tissues after salt treatments. (**a**) Leaf discs detached from transgenic plants overexpressing the *TgERF1* and WT plants were floated on MS liquid medium supplied with 0, 400, and 800 mM NaCl for 3 days. Bar: 0.5 cm. (**b**) Chlorophyll index (SPAD-value) in leaf tissues after salt treatment. In each experiment, 15 leaf discs/line were used. Bars represent mean value ± SEM. Significant differences were determined by ANOVA followed by Dunnett’s test. ** Significant at *p* < 0.01.

Considering that salt treatments significantly induced *TgERF1* expression in transgenic tobacco during the post-germinative growth, we investigated whether *TgERF1* overexpression could increase salt tolerance in transgenic tobacco. To assess the salt stress tolerance capabilities at the tissue level, leaf discs of transgenic and WT tobacco plants were separately floated on MS liquid medium supplemented with 0, 400, or 800 mM NaCl. At three days of salt treatment, the damage caused by stress was obvious in the degree of bleaching observed in the leaf tissues (Figure 8a). Whilst the leaf discs from transgenic *TgERF1* plants remained green, leaf discs from the WT were bleached. Chlorophyll index (SPAD-value) measurements in these plants confirmed phenotypic differences (Figure 8b). Under NaCl treatments, the total chlorophyll losses in WT discs compared with L1, L2, and L3 lines of the transgenic plants were around 93, 77, 44, and 36%, respectively.

### 2.8. Role of TgERF1 under Drought Stress

**Figure 9:**
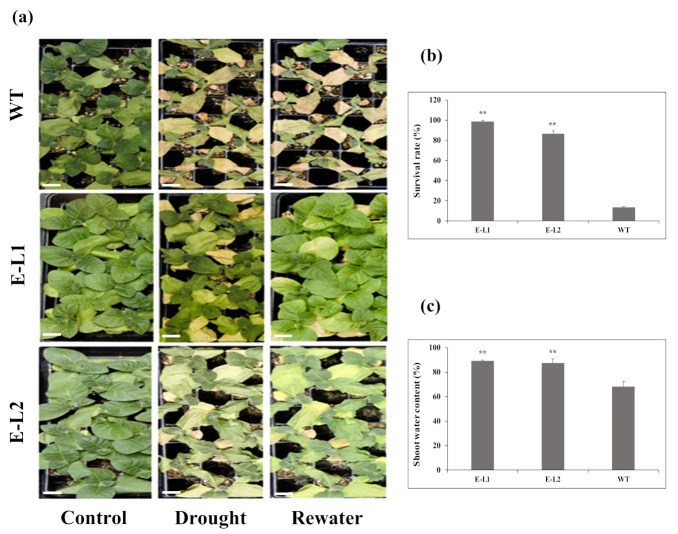
Phenotypic differences among *TgERF1* transgenic tobacco and WT under drought stress. (**a**) Phenotype of transgenic and WT plants initially grown for three weeks under usual conditions, which was followed by 12 days under drought stress and then by rewatering for three days. Bar: 2 cm (**b**) Survival rates and (**c**) shoot water content was measured after 4 days of drought stress recovery. Bars show mean value ± SEM. Significant differences were determined by ANOVA followed by Dunnett’s test. ** Significant at *p* < 0.01.

For the evaluation of *TgERF1* capability to enhance drought tolerance, 2-week-old tobacco seedlings were transferred to trays containing a soil mixture of vermiculite and humus (1:2). The 3-week-old tobacco seedlings were then grown under severe drought stress and usual well-watered conditions (Figure 9a). Since L1 and L2 transgenic lines showed significant stress responses they were chosen for analyses. At 12 days without watering, wilting symptoms were observed in L2 and WT plants, whereas L1 leaves displayed less wilting at visual observation (Figure 9). After rehydration for three days, most of the WT plants died (survival rate of 13.1%), but transgenic plants displayed drought tolerance and recovered rapidly (survival rates of 89%, 87.3% for L1 and L2, respectively; Figure 9b). After rehydration, we also found that water content in *TgERF1* L1 was 89%, and L2 was 87.3%. These results were significantly different from the 67.3% water content of WT plants (Figure 9c). Therefore, the results demonstrated that the transgenic plants had better water retention ability under drought stress conditions and could increase drought tolerance in tobacco.

### 2.9. Physiological Changes of Tobacco Transgenic Lines Overexpressing TgERF1

**Figure 10:**
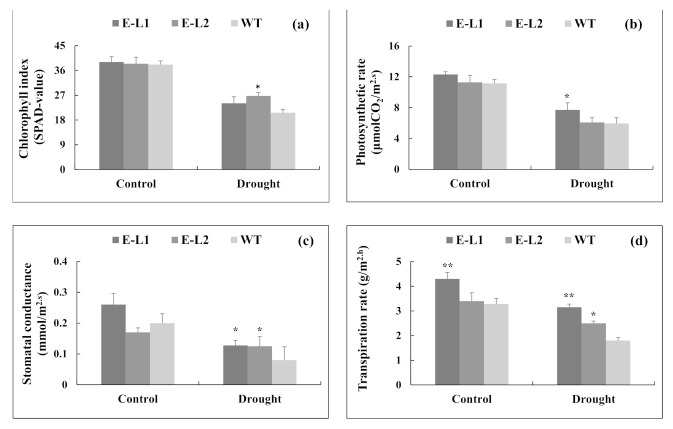
Chlorophyll index (SPAD-value) and photosynthetic parameters of *TgERF1* transgenic tobacco and WT in response to drought stress. Plants were grown in individual pots and were watered with one-fifth-strength Hoagland solution and weighed daily during the experiment. Well-watered control plants were grown in 100% field capacity (0% of water loss). The time-course drought stress assay started by withholding the nutrient solution until reaching 70% water loss. The (**a**) chlorophyll index, (**b**) transpiration rate, (**c**) stomatal conductance, and (**d**) photosynthetic rates were measured in leaves of transgenic plants and WT plants under well-watered and water-deficit conditions. Data are presented as the means ± SEM of four biological replicates. Significant differences were determined by ANOVA followed by Dunnett’s test. ** Significant at *p* < 0.01 and * at *p* < 0.01.

Data summarized above suggested that *TgERF1* overexpression resulted in an improved response of transgenic plants to stress treatments. Therefore, the *TgERF1* overexpression may have caused a concerted stimulation of biochemical and physiological processes that regulate plant vigor. This hypothesis was tested by analyzing several representative physiological parameters in an experiment conducted with plants grown in individual pots. Chlorophyll index (SPAD-value), photosynthesis (A), stomatal conductance (Gs), and transpiration rate (E) decreased proportionally from the control to drought stress conditions in all plants (Figure 10a–d). Under drought conditions, there was no significant differences in chlorophyll indexes (SPAD-value) between the L1 transgenic line and WT plants (Figure 10a), whereas the L2 transgenic line showed the highest values (22%) compared to WT plants. However, the L1 line displayed the highest value of photosynthetic rate when drought stress was applied, which was 24% higher than WT plants (Figure 10b). Under drought stress, Gs and E parameters were increased in all transgenic lines compared to WT plants (Figure 10c,d). The transpiration response of the L1 line was significantly higher than the L2 Line and WT plants in both control and stress conditions. Moreover, under control and drought conditions L1 plants maintained higher photosynthetic rates and Gs values in comparison to WT plants.

**Figure 11:**
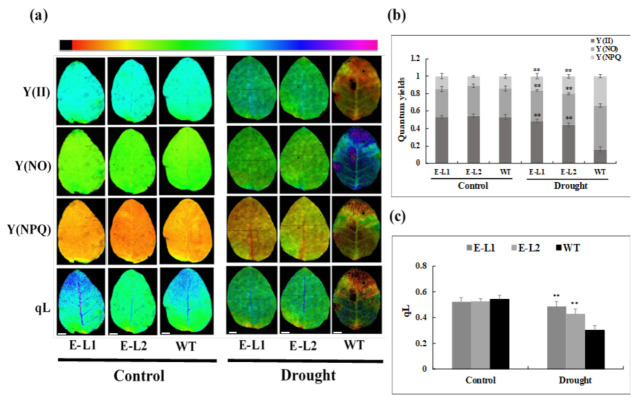
Chlorophyll fluorescence parameters in *TgERF1* transgenic and WT plants under water-deficit treatment (Drought) and under well-watered conditions (Control). Plants were grown in individual pots, watered with one-fifth-strength Hoagland solution, and weighed daily during the experiment. Well-watered control plants were grown in 100% field capacity (0% of water loss). The time course drought stress assay started by withholding the nutrient solution until reaching 70% water loss. (**a**) Chlorophyll fluorescence images of Y(II), Y(NO), and Y(NPQ) are shown. The color scale at the top represents absolute values ratio ranging from 0 (black) to 1.0 (pink). Bar: 1.5 cm (**b**) Quantum yield of PSII, including Y(II) (actual photosynthetic efficiency of PS II), Y(NO) (quantum yield of non-regulated energy dissipation), and Y(NPQ) (effective quantum yield of PS II). (**c**) Changes in qL (photochemical quenching coefficient). Physiological parameters were registered in leaves of transgenic and WT plants under normal and water-deficit conditions. Data are presented as the means ± SEM of four biological replicates. Significant differences were determined by ANOVA followed by Dunnett’s test. ** Significant at *p* < 0.01.

Regarding chlorophyll fluorescence parameters, levels were similar for the wild-type and *TgERF1* transgenic plants under usual growth conditions (Figure 11a). However, under drought stress the actual photosynthetic efficiency (Y(II)) and the coefficient of actinic light quenching (qL) significantly declined in leaves of WT plants, and they reached approximately 66% and 37%, respectively (Figure 11b,c). Under drought, quantum yields of non-regulated energy dissipation Y(NO) and quantum yield of regulated energy dissipation (Y(NPQ)) declined significantly in both transgenic lines compared to WT plants, approximately 43% and 85%, respectively (Figure 11b,c). Based on the results shown in Figure 10 and Figure 11, we suggest that overexpression of *TgERF1* improved the physiological conditions against damage processes in transgenic tobacco plants under drought stress.

**Figure 12:**
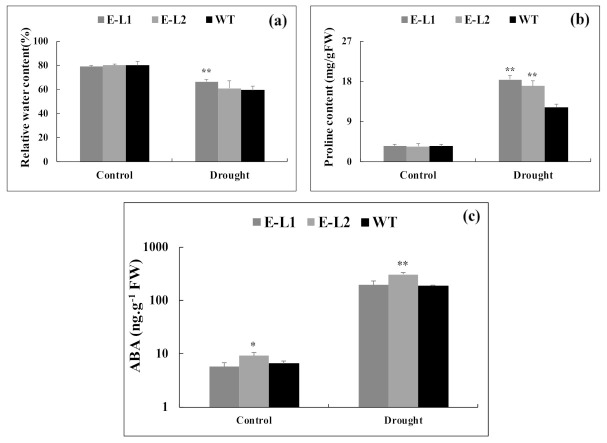
*TgERF1* transgenic and WT tobacco plants in response to drought stress. (**a**) Relative water content. (**b**) Proline. (**c**) Endogenous ABA concentration. Plants were grown in individual pots, watered with one-fifth-strength Hoagland solution and weighed daily during the experiment. Well-watered control plants were grown in 100% field capacity (0% of water loss). The time course drought stress assay started by withholding the nutrient solution until reaching 70% water loss. Leaf contents of proline, relative water, and endogenous ABA were measured in transgenic and WT plants under drought and control conditions. Proline was quantified spectrophotometrically. Data are presented as means ± SEM of four biological replicates. Significant differences were determined by ANOVA followed by Dunnett’s test. ** Significant at *p* < 0.01 and * at *p* < 0.01.

To test whether the increased expression of *TgERF1* caused changes in leaf water content, the relative water content (RWC%) was measured in transgenic and WT plants (Figure 12a). RWC after drought stress in both WT plants and *TgERF1*-overexpressing plants decreased, but the levels were significantly higher in L1 line plants than in WT. To investigate the underlying mechanisms of the recovery phenotype of 35S:ERF1, we determined the proline content and endogenous hormones on leaves (Figure 12b,c and Figure S5). Under normal growth conditions, free proline concentrations in *TgERF1* transgenic tobaccos and WT plants were similar. During drought treatment, contents of proline in both *TgERF1* transgenic lines and the control plants increased continuously, but in the transgenic plants they were significantly higher than those in the WT plants (Figure 12b). The endogenous accumulation of ABA was already significantly higher in *TgERF1*-L2 transgenic line under control conditions. Although drought treatment caused a 10-fold increase in ABA accumulation in transgenic and control plants, only the L2 line had a significantly higher accumulation of ABA compared to WT plants (Figure 12c).

Concentrations of other hormones varied among genotypes in both conditions. Drought stress reduced SA, JA, and iP concentrations in all transgenic lines compared to control plants (Figure S5a–c). GA1 levels were considerably increased in L1 plants (Figure S5d). In addition, in the control condition, endogenous JA and iP levels were higher in transgenic lines compared to WT plants. As for the stress treatment, levels of these two hormones increased in both transgenic lines and in control plants.

### 2.10. Expression of Stress-Responsive Genes in TgERF1-Transgenic Plants

**Figure 13:**
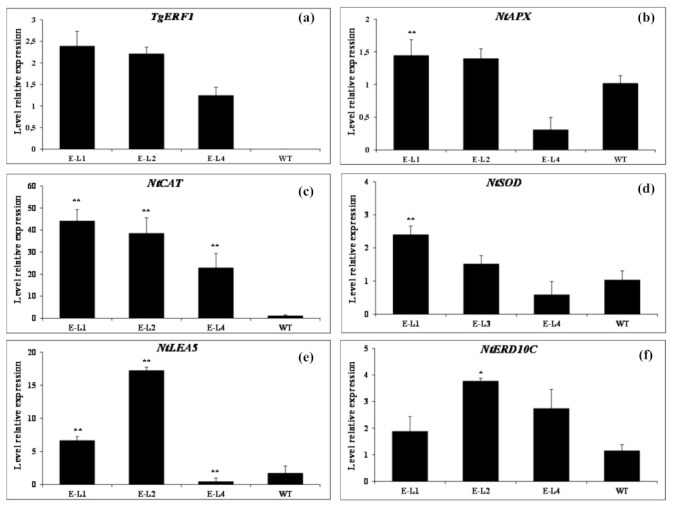
Relative expression of stress responsive genes at ten days of drought treatment. (**a**) Characterization of expression levels of the *TgERF1* transgene. (**b**–**f**) Stress responsive genes *NtAPX*, *NtCAT*, *NtSOD*, *NtLEA5*, *NtERD10C*. Total RNA was isolated from plant leaf tissues at ten days of drought stress. Measured parameters were performed at 40 days after sowing (DAS). The constitutive *NtEF1α* housekeeping gene was used as internal control. Data are presented as means ± SEM of three biological replicates. Significant differences were determined by ANOVA followed by Dunnett’s test. ** Significant at *p* < 0.01 and * at *p* < 0.01.

The results above showed that overexpression of *TgERF1* may play an important role in the regulation of gene expression in response to drought stress. A new assay was carried out aiming to better understand the regulatory role of *TgERF1* at a transcriptional level during drought stress. The RT-qPCR was used to assess expression levels of the *TgERF1* gene in conjunction with five representative stress-responsive genes in the 35S::TgERF1 lines and WT plants under the drought treatment, including four antioxidant-related genes in tobacco *NtAPX* (ascorbate peroxidase), *NtCAT1* (catalase 1), *NtSOD* (superoxide dismutase), and *NtERD10C* (early responsive to dehydration 10C), and *NtLEA5* (late embryogenesis abundant 5) genes (Figure 13). After drought stress, relative expression levels of *TgERF1* varied in the three transgenic lines. Compared to L4, the *TgERF1* expression was higher in the L1 and L2 lines, at values of 1.92- and 1.78-fold, respectively (Figure 13a). As for the *NtAPX* gene, mRNA transcript levels were significantly higher in the L1 and L2 lines than in WT plants (Figure 13b), while a slight difference was observed in transcript levels of the L4 line, approximately two-fold less than WT plants. Regarding the *NtCAT* gene, under drought stress there was a striking elevation of this gene expression in L1, L2, and L4 transgenic plants compared to WT, at approximately 43-, 37-, and 21-fold higher, respectively (Figure 13c). Transcript levels of the *NtSOD* gene underwent minor changes in transgenic lines in comparison with WT plants and with the greatest significant increase observed in the L1 line (Figure 13d). The *NtLEA5* and *NtERD10C* expression in transgenic plants had a different pattern (Figure 13e,f). For *NtLEA5* and *NtERD10C* genes, the expression levels in the L2 line showed a significant increase compared to WT plants. These results indicate that overexpression of *TgERF1* can participate in the drought stress response possibly through indirectly controlling transcriptional regulation of stress-responsive genes.

## 3. Discussion

The AP2/ERF family has been widely studied in several plant species. Despite this, the present study is one of the first reports on the identification and characterization of AP2/ERF transcription factors in the Lamiaceae taxonomic group. Molecular and physiological data on the identified *TgERF1* TF from the *Tectona grandis* genome contribute to a better understanding of the AP2/ERF family and its role in this wood species and on plant stress tolerance. The AP2/ERF domain proteins belong to AP2 and EREBP subfamilies, in which genes probably emerged before the divergence of the Chlorophyta lineage from the Streptophyta lineage. A few genes of this subfamily are found in Chlorophytas (approximately five depending on the species) [33]. In teak, 201 AP2/ERF proteins of this family were identified, and this high number is probably due to several gene duplication events. It is known that the evolutionary trajectory of AP2/ERF duplication may undergo two stages: post-duplication functional divergence, followed by a generally slow evolutionary rate, owing to the high level of functional constraints after functional divergence [34,35]. These genetic duplication events helped to strengthen plant species’ responses to adverse environmental factors by generating newly sub-functionalized genes, which facilitated a more precise regulation of signaling networks and adaptation [32]. This regulation is due to the AP2/ERF ability to bind to several cis-elements present in promoter regions of stress-responsive genes [36].

In general, AP2/ERF plays significant roles in regulation of genes and is tightly involved in plant responses to various adverse environmental conditions in Arabidopsis [37], cotton [38], and rice [39]. The ERF and DREB subfamilies identified in our work have two subdivisions located in two contiguous branches, DREB a and b, and ERF a and b (Figure 1), but both are evolutionarily close, as in the classification reported in [40,41]. Notably, this repeated pattern indicates that the expansion of ERF and DREB subfamilies possibly have occurred from the AP subfamily. Motifs 1–3 were found to be part of the AP2/ERF domain region, while the remaining motifs corresponded to regions outside of the AP2/ERF domain region, which are distributed in specific clades in the teak phylogenetic tree (Figure 2a,b).

Numerous studies have suggested that transcription factors are modulated by mechanisms involving their synthesis, subcellular localization, DNA binding, and other posttranslational mechanisms [42]. In our study, the subcellular localization analysis and computational predictions indicated that the TgERF1 protein is localized in the cell nucleus (Figure 5). Previous studies reported that the ERF38 protein encoded by the *ERF38* (Potri.006G138900.1) gene from the 84K poplar hybrid (*Populus alba* × *Populus glandulosa*) was targeted to the nucleus and had no self-activation [43]. In addition, the sweet potato IbRAP2-12 protein is localized in the nucleus as revealed by the transient expression in tobacco epidermal cells [16]. Similarly, the maize ZmEREB160 protein is also localized in the nucleus and has transcription activation activity [44].

The ERF subfamily has been reported as one of the largest subfamilies of transcription factors, with a significant regulatory role on plant growth, developmental response, and stress tolerance [45]. AP2/ERFs are plant-specific transcription factors [2]. In teak tissues, RT-PCR analysis demonstrated maximum *TgERF1* gene expression in leaves, followed by roots, and then stems (Figure S1). In teak leaves, *TgERF1* expression was induced by PEG, MeJA, and ABA treatments. NaCl salt stress treatments induced the lowest increase in *TgERF1* expression. Expression induction patterns of the *TgERF1* gene were similar to the *TaERF1* from Caucasian clover, which is induced by multiple environmental stresses and exogenous hormones such as drought, salt, ABA, SA, and ET [46]. Moreover, the expression of the soybean *GmERF3* gene was induced by high-salinity stress, drought, ABA, JA, SA, and ET [29], whereas *CaPF1* expression in hot pepper is induced by ethephon, MeJA, cold, and NaCl treatment [47]. In poplar, the expression of the *ERF76* gene is induced under high salinity stress [48].

Recent studies have suggested that hormone signaling pathways regulated by ABA, SA, JA, and ethylene play key roles in the crosstalk between biotic and abiotic stress signaling [7]. Therefore, we hypothesized that *TgERF1* may be involved in plant stress tolerance. Several studies have demonstrated that AP2/ERF TFs can enhance stress tolerance when overexpressed in transgenic plants. For instance, overexpression of the rice *JERF1* gene significantly increases the growth of tobacco roots and leaves under salinity and low-temperature treatments [49]. Similarly, overexpression of *ERF1-V* enhances powdery mildew, salt, and drought stress tolerance of transgenic *A. thaliana* [50]. Overexpression of *Tsi1*, an AP2-type transcription factor, significantly enhanced tolerance to osmotic stress in tobacco [36]. Moreover, it has been reported that overexpression of the pepper *CaPF1* gene in pine (*Pinus virginiana* Mill.) enhanced drought stress tolerance by changing the morphological structure of the leaves and increasing cell numbers [27].

Therefore, the function of *TgERF1* was further examined in transgenic tobacco plants constitutively overexpressing the *TgERF1* protein and exposed to drought and salt stress. Transgenic plants overexpressing *TgERF1* showed a normal phenotype, but seedling growth ratios increased significantly in transgenic plants exposed to PEG, mannitol, and NaCl stress conditions, compared to WT plants. Growth increase was also reported in previous findings for tobacco seedlings overexpressing the *GmERF3* gene under diverse abiotic stresses [29].

Considering the results, tobacco L1 and L2 homozygous transgenic lines overexpressing the *TgERF1* transgene were further investigated attempting to better understand the role of this teak TF in salinity and drought stress tolerance. The chlorophyll index (SPAD-value) was significantly higher in L1-L2 seedlings than in WT plants under 400 mM and 800 mM NaCl treatments. High chlorophyll index (SPAD-value) in transgenic tobacco plants has been correlated with improved tolerance to salinity stress [51]. Higher chlorophyll retention capacity by transgenic lines over WT plants under salt stress was also reported for tobacco plants overexpression of the soybean GmERF3 TF [29]. Under stress conditions, seedlings overexpressing *TgERF1* exhibited better phenotypic morphology after rewatering, and they were more tolerant to drought stresses compared to the WT plants. Similarly, the overexpression of other ERFs TFs in Arabidopsis such as *IbRAP2-12* [16] and *AtERF1* [7] conferred enhanced abiotic stress tolerance over WT in seedlings.

The data summarized above motivated additional analysis aiming to identify representative physiological parameters that could be related to the increased tolerance of *TgERF1* transgenic plants to stress. Regarding plants at 70% water loss under drought stress, the A, Gs, and E values decreased less in transgenic lines than in the WT control (Figure 12). The change in the Gs trend was similar to the one observed in A and E between WT plants and overexpressed lines, which indicated that the high A and E values in transgenics lines were dependent mainly on Gs regulation [52]. A similar result was described when the AP2/ERF family gene from Arabidopsis was overexpressed in sugarcane plants under water deficit. These transgenic plants were able to maintain higher physiological parameters compared to the WT plants without biomass penalty [53]. Furthermore, overexpression of *HARDY*, an AP2/ERF gene from *Arabidopsis*, resulted in enhanced drought stress tolerance in rice by reducing transpiration and improving photosynthesis [26]. An opposite result was observed when *SIERF36* was overexpressed in tobacco and, in this case, the transgenic lines showed lower A, Gs, E, and stomal density compared to WT plants. In addition, there was a further reduction in CO_2_ assimilation rates and growth, early flowering, and senescence in these tobacco transgenic plants [15].

The chlorophyll index and chlorophyll fluorescence parameters of leaves are indicators of the photosynthetic capability of plant tissues under environmental stress [23]. The effectiveness of photosystems II can be reflected by chlorophyll fluorescence indexes, which represent the energy distribution and the activity of the photosynthetic reaction center in PS II [54]. The present study has shown that drought stress affected Y(II), Y(NO), Y(NPQ), and qL, which suggested that drought stress reduced the quantity of light quantum absorbed by the reaction center and shutdown PS II. The reason behind the enhancement of light harvesting efficiency under drought stress in transgenic lines is due to the heightening of the chlorophyll index (Figure 13a). The high Y(NO) in WT plants under drought stress indicated an increase in heat dissipation unregulated and weakening of photosynthetic efficiency [55]. The Y(NPQ) represents heat dissipation as a plant mechanism of photo-protection, state transition, and photo-inhibition [56,57]. It has been described that Y(NPQ) increases under stress conditions such as high light intensity or photoinhibition, salinity, heavy metal toxicity, drought, or chilling [58]. Therefore, *TgERF1* transgenic plants maintained lower amounts of Y(NPQ) compared to WT plants, which could probably mean that the Y(NPQ) gene helps regulate the heat dissipation and photo-protective mechanisms, through the improved absorption and transmission of light energy.

The *TgERF1* transgenic plants had higher RWC values than the WT plants. Regarding the apparent disagreement between the higher RWC in transgenic plants shown in Figure 12a, and the higher transpiration rate (E) and Gs shown in Figure 10c,d, we suggest that transgenic plants can absorb and retain more water by osmotic adjustment under water stress conditions [59]. Accumulating evidence has indicated that plants adapting to abiotic stress conditions can induce the production of various osmolytes in plant cells [60,61]. Proline is known to be an important osmoprotective solute that contributes to osmotic adjustment, protects enzymes, and stabilizes macromolecules and membranes in cells exposed to stress [62]. Therefore, proline contents were measured to examine the role of *TgERF1* in osmotic adjustment and in membrane protection. The overexpression of *TgERF1* led to increased proline in the transgenic lines compared to WT plants. The increased levels of proline in the plants overexpressing *TgERF1* indicate that this transcription factor participates in the regulation of this important substance that prevents dehydration under abiotic stress. It is possible that *TgERF1* participates in signaling pathways that regulate the expression of stress-responsive genes under drought.

Phytohormones have different functions in the growth, development, and environmental adaptation in plants [63]. Previous studies have shown that several AP2/ERF transcription factors regulate the expression of genes from ABA-dependent pathways [7,23]. Consistent with that observation, we showed here that ABA accumulation in the *TgERF1* L2 line was significantly higher than in WT plants under drought stress, although the L1 line showed no significant difference to the wild-type plants (Figure 12c). Furthermore, the L2 line appears to increase ABA production in the absence of stress, which may initially help to protect against stress [64]. As noted by the authors of [59], overexpression of *ORA47*, an AP2/ERF gene in *Arabidopsis*, activated ABA and JA biosynthesis and signaling genes under stress conditions, such as *DAD1*, *NCED3*, *NCED9,* and *MYC2* genes. *JERF1* overexpression increased levels of endogenous ABA whereas WT tobacco did not show differences under normal growth conditions. However, the mechanism by which *JERF1* mediates ABA action and biosynthesis remains unknown [51]. These results suggest that *TgERF1* affects the ABA signaling pathway in tobacco plants.

Drought and salt stresses cause a rapid accumulation of reactive oxygen species (ROS), resulting in oxidative stress and cell damage [65]. Plants have developed an elaborate and antioxidant system to eliminate these toxic compounds, leading to a positive correlation between abiotic stress tolerance and the activity of ROS-scavenging antioxidant enzymes [63]. In order to understand the regulatory function of *TgERF1* and to explain the enhanced drought tolerance at molecular levels, RT-qPCR was carried out to quantify transcription levels of *NtAPX*, *NtCAT*, *NtSOD*, *NtLEA5,* and *NtERD10C*. All five genes were significantly upregulated through *TgERF1* overexpression in L1 and L2 lines subjected to drought stress (Figure 13). A higher induction of ROS-scavenging genes such as ascorbate peroxidase (*NtAPX*), catalase 1 (*NtCAT1*), and superoxide dismutase (*NtMnSOD*) was observed in the L1 and L2 lines as compared to WT plants. This result can presumably explain the increased stress tolerance capabilities of these transgenic lines. *NtLEA5* and *NtERD10C* have pivotal roles in maintaining cell homeostasis [66,67]. The high induction of both genes provides more protective macromolecules that maintain membrane integrity during drought stress [66,67,68]. The increased levels of several stress-inducible genes in the transgenic lines indicated that *TgERF1* may participate in stress-responsive signaling pathways (Figure S7), positively regulating the transcription of these genes.

## 4. Materials and Methods

### 4.1. Database Search and Phylogenetic Analysis of AP2 Transcriptional Factors

AP2 proteins were already identified from proteomes deduction accessed in the Dryad database for teak (Figure S6) [69], ARAPORT11 for *Arabidopsis thaliana* (Arabidopsis) [70], and JGI v7.0 for *Oryza sativa* sp. Japonica (rice) [71]. Any protein carrying the AP2 domain was considered a member of the TF AP2 family of TFs. The AP2 domain was searched and identified using the profile Hidden Markov Model for this domain from PFAM (Accession Number PF00847). This profile was downloaded from Pfam Database (http://pfam.sanger.ac.uk/ (accessed on 17 November 2019)) and a hmm-search was performed with the HMMer software v3.2.1 (Cambridge, MA, USA) ‘hmmscan’. Hit scores higher than 26.6 (gathering cutoff from PFAM specific for the AP2 model) were considered true positives and selected for further analyses. AP2 proteins from *Arabidopsis* (184), *Oryza sativa* sub species japonica (135) and *Tectona grandis* (201) were aligned using MAFFT v7.407 in the auto mode. Regions of low quality from the multiple sequence alignment were removed using TrimAl v1.4, in the “automated1” mode [72] from Phylemon2 [73]. Phylogenetic inference under the Maximum Likelihood approach was carried out with IQ-Tree v1.6.9, with the options “-m MFP -st AA -seed 12,345 -lmap 65,400 -alrt 1000 -bb 1000”. The best evolutionary model for phylogenetic inference was also estimated within IQ-Tree with the -m MFP option (Model Finder Plus), by computing the log-likelihoods for many different evolutionary models against an initial parsimony tree and choosing the model that minimizes the Bayesian information criterion (BIC). Branch support was assessed with the Shimodaira–Hasegawa (SH)-like approximate likelihood ratio test and ultrafast bootstrap [74], both with 1000 replicates. The subfamilies were classified according to [32]. The subfamily containing the TgERF1 protein was aligned using MAFFT v7.407 in the auto mode, and the phylogenetic analysis was performed with IQ-Tree v1.6.9. The structure of AP2 domain protein was performed by detection of conserved motif with software MEME v5.0.5, and the following parameters: distribution of motif occurrences, zero or one per sequence; minimum width, 6, maximum width, 50; maximum number of motifs, 10; and optimum motif width, ≥6 and ≤24 [75].

### 4.2. Plant material and Growth Conditions

Sixteen-year-old *Tectona grandis* trees grown in an experimental field of the College of Agriculture Luiz de Queiroz, University of Sao Paulo, Piracicaba, São Paulo State, Brazil (Latitude: 22°42′23″ S, Longitude: 47°37′7″ W, 650 m above sea level), were used to isolate the full-length coding DNA sequence (CDS) of the *TgERF1* gene (*Tectona grandis*) according to [8,30].

Teak experiments for analysis of *TgERF1* tissue-expression patterns in stems, leaves, and roots, as for stress treatments, were completed using shoots from two-month-old teak seedlings grown in glass flasks onto 30 mL half-strength MS culture medium (Murashige and Skoog; Sigma-Aldrich, St. Louis, MO, USA) supplemented with 3% sucrose and 0.8% agar, pH adjusted to 5.8. They were incubated at 25 °C under 16 h light/8 h dark photoperiod, adjusted to 45 μmol photons m^−2^ s^−1^ PAR irradiance.

Seeds of *Nicotiana tabacum* L. cv. Petit Havana were surface sterilized for 1 min with 70% ethanol and then treated with commercial bleach (2.7% sodium hypochlorite) diluted with water (1:2 v/v) for 10 min. They were then rinsed four times with distilled water. Seeds were germinated onto 30 mL culture medium contained in glass flasks, which consisted of half-strength MS medium (Murashige and Skoog; Sigma-Aldrich, St. Louis, MO) supplemented with 3% sucrose (pH 5.8, 0.8% agar). They were incubated at 25 °C under long-day conditions (16 h light/8 h dark) adjusted to 45 μmol photons m^−2^ s^−1^ PAR irradiance.

### 4.3. Teak Stress Treatments

For analysis of *TgERF1* tissue-expression patterns, sampled stems, leaves, and roots were independently harvested and immediately frozen in liquid nitrogen and stored at −80 °C until RNA extraction. To induce stress treatments, 2-month-old seedlings were transplanted to a fresh MS culture medium supplemented with different concentrations (150 mM) of NaCl (high salinity), 20% PEG 4000 (high osmotic pressure), 100 μM MeJA (Bedoukian Research Inc., Danbury, CT, USA), 100 μM ABA (Sigma-Aldrich), or 100 mM ethylene precursor ACC (1-aminocyclopropane-1-carboxylic acid) (Sigma-Aldrich). Leaf samples were collected at 0 h, 3 h, 6 h, and 12 h after treatment, and frozen immediately in liquid nitrogen.

### 4.4. RNA Extraction and RT-PCR and RT-qPCR of Teak

Total RNA was isolated from teak tissues using the TRIzol reagent (ThermoFisher, USA) following the manufacturer’s instructions. Genomic DNA contamination in total RNA samples was eliminated by RNAse-free DNAse I (Promega, Madison, WI, USA), and cDNA was synthesized using the SuperScript™ III First-Strand Synthesis System from 1 μg of total RNA according to the manufacturer’s protocol (ThermoFisher, Waltham, MA, USA).

As for RT-PCR, *TgERF1* primers (TgERF1RT_F and TgERF1RT_R, Table S3) were used for the *TgERF1* target gene. The constitutive *Elongation Factor-1 alpha* (*TgEF-1 alpha*) housekeeping gene was used as an internal control with TgEF-1AF and TgEF-1AR primers (TgEF-1AF and TgEF-1AR, Table S3). PCR cycling conditions consisted of an initial incubation at 95 °C for 5 min, followed by 40 cycles of 95 °C for 15 s, 60 °C for 30 s, and 72 °C for 30 s. For the *TgERF1* expression analysis, RT-qPCR was performed on an ABI 7500 qPCR thermocycler (Applied Biosystems, Waltham, MA, USA) with the Platinum SYBR Green Supermix (Invitrogen, Waltham, MA, USA). PCR cycling conditions consisted of an initial incubation at 95 °C for 10 min, followed by 40 cycles of 95 °C for 15 s, 60 °C for 30 s, and 72 °C for 30 s. The *TgEF-1 alpha* housekeeping gene was used as an internal control (Table S3), and the target gene was *TgERF1* (Table S3). The ΔΔCt method was used to analyze the relative expression level in the real-time PCR data [76]. Each measurement was carried out with three biological replicates, and two technical replicates.

### 4.5. TgERF1 Overexpression Vector Construction and Plant Transformation

The full-length coding sequence (CDS) of *TgERF1* (NCBI accession: MH003850, Table S7) was PCR-amplified using template cDNAs from teak stem according to [30]. PCR primers were designed containing the “CACC” sequence in the N-terminal region (TgERF1CDSF), according to the manufacturer’s instructions for the pENTR™/D-TOPO^®^ cloning vector (Thermo Fisher, Carlsbad, CA, USA), and the ‘TGA’ stop codon from the amplicon was excluded (TgERF1CDSR) to allow the expression of the reporter gene (*EGFP*) (Table S4). The PCR product was cloned into pENTR^™^/D-TOPO^®^ cloning vector (Thermo Fisher, Carlsbad, CA, USA) according to the manufacturer’s instructions, and then transformed into *Escherichia coli* cells (DH5α) and validated by Sanger sequencing. The overexpression vector was obtained using multiple recombinations among the pENTR (containing *TgERF1*) and binary vector pK7FWG2 (Life Technologies, Carlsbad, CA, USA) using the Gateway LR Clonase II Enzyme Mix (Thermo Fisher, Carlsbad, CA, USA). The pK7FWG2 vector contained the neomycin phosphotransferase II (*NPTII*) selective marker gene and the enhanced green fluorescent protein (*EGFP*) reporter gene, both genes being under the control of the cauliflower mosaic virus (CAMV35S) promoter (Figure S8). The position and integrity of the transgene in the vector were confirmed by digestion with restriction enzymes, gel electrophoresis, PCR, and Sanger sequencing. The resulting construct containing p35S::TgERF1 was named 35S::TgERF1 and used to transform *Agrobacterium tumefaciens* strain EAH105 by electroporation.

Tobacco leaf discs from 30-day-old tobacco plants were transformed via *Agrobacterium tumefaciens* with the 35S::TgERF1-EGFP vector, using a standard protocol. The pK7FWG2 empty vector was used as a control. The transformed explants were kept at 25 ± 1 °C, in the dark, for 2 days. After this period, the explants were transferred to MS agar medium supplemented with 1 mg L^−1^ BAP, 100 mg L^−1^ kanamycin (Sigma, Saint Louis, MO, USA) and 300 mg L^−1^ Timentin (Sigma, Saint Louis, MO, USA) and incubated at 25 °C, under 16 h light/8 h dark photoperiod for 3 weeks. Responsive explants (2–4 mm) were transferred to glass pots (95 × 65 mm) containing 30 mL MS agar medium supplemented with 0.1 mg L^−1^ NAA, 100 mg L^−1^, kanamycin, and 300 mg L^−1^ Timentin and incubated for 3 weeks for rooting. Putative transgenic true shoots were confirmed by PCR using the 35S promoter (p35SPCRF) and the *TgERF1* (TgERFCDSR) primers, and by RT-PCR using the *TgERF1R* primers (Table S5). Shoots regenerated from a single callus were treated as a single transformation event (Lines). At three weeks of culture, plants were analyzed for developmental pattern and transferred to the greenhouse in pots (3 L) with commercial substrate Basaplant^®^ (Base Agro, Artur Nogueira, SP, Brazil), supplemented with 1 g NPK 10:10:10 L^−1^. The T0 seeds were collected and germinated on MS medium with kanamycin (100 mg L^−1^) to produce the T1-generation transgenic progenies. Transgenic T1 tobacco plants were transferred to the greenhouse to generate T2 progenies. Positive transgenic plants were screened for kanamycin resistance. In all experiments, WT controls consisted of plants cultivated in vitro and not treated for transformation. Phenotypic and molecular characterizations were performed on T2 plants of homozygous transgenic lines and WT plants.

### 4.6. Measurement of Growth Parameters

Root growth rates were estimated by measuring primary root length of vertically grown seedlings from hypocotyl base to root tip, at 7 days, using Image J version 1.45 (National Institutes of Health; http://rsb.info.nih.gov/ij/ (accessed on 20 February 2020)). A total of 20 seedlings were measured for each transgenic line and WT plants distributed in 4 replicates. Mean data were subsequently analyzed using ImageJ version 1.45 to estimate root elongation.

### 4.7. Subcellular Localization of the TgERF1 Protein

Fluorescence visualization was analyzed in the root differentiation zone of 7-day-old T1 plants using a confocal microscopy FV1000 Olympus FluoView (Olympus, Tokyo, Japan) with an argon laser. The excitation wavelength was calibrated between 488 and 509 nm. The subcellular localization was predicted by using the online WoLF PSORT software (https://wolfpsort.hgc.jp/ (accessed on 25 August 2020)), and the HMMTOP software was used for transmembrane prediction.

### 4.8. RNA Extraction and RT-PCR of Transgenic Tobacco

Total RNA was isolated from 10-day-old tobacco leaf tissues, and cDNA synthesis was performed as previously described (4.4). The constitutive *Elongation Factor- 1 alpha* (*NtEF-1 alpha*) housekeeping gene was amplified as an internal control using primers NtEF-1AF and NtEF- 1AR [77] (Table S5), and *TgERF1* was the target gene using primers TgERF1RT_F and TgERF1RT_R (Table S5). Cycling conditions were 95 °C for 5 min, followed by 35 amplification cycles consisting of 95 °C for 15 s, 60 °C for 30 s, and 72 °C for 30 s.

### 4.9. Analysis of TgERF1 Transgenic Plants under Osmotic and Saline Stresses

#### 4.9.1. Root Growth Assay

Root length assays under abiotic stress were carried out with WT control and three selected 35S::ERF1 transgenic lines named L1, L2 and L4. Three-day-old were transferred to MS agar medium supplemented with 0,5%, 10%, or 20% PEG 4000 (w/v); 0, 100, 200, or 300 mM mannitol; and 0, 50, 150, or 200 mM NaCl and grown for seven days. All plates were placed in the upright position in the growth chamber. The primary root length and lateral root numbers were analyzed as described above (4.6). Root growth was measured at seven days of treatment in three independent experiments, with 48 individuals per genotype in each experiment. Thereafter, the best lines were selected for stress tolerance studies.

#### 4.9.2. Leaf Discs Assay

The leaf disc assay under salt stress experiment was prepared by using the third leaf counted from the top of one-month-old plants of WT, and two lines 35S::ERF1 genotypes. Leaf discs of 0.7 cm diameter were punched using a cork borer and placed on MS liquid medium supplemented with 0, 400, and 800 mM NaCl for three days. They were incubated under 16 h light/8 h dark photoperiod, at 25 °C. The chlorophyll index was estimated with the leaf-clip chlorophyll meter SPAD-502 (Konica Minolta, Inc., Tokyo, Japan). The experiment was carried out in triplicate and measured at least 15 leaves for each genotype in each replicate.

### 4.10. Plant Survival under Drought Stress

Survival rates under drought stress were assessed in tobacco seedlings of L1 and L2 transgenic 35S::ERF1 lines and the wild type used as a control. Two-week-old seedlings were transferred to trays containing a soil mixture consisting of vermiculite and humus (1:2) and grown in a phytotron. Three-week-old plants were than subjected to severe drought stress by withholding water for 12 days followed by rewatering for three days. After the recovery period, the surviving plants were photographed, leaves were manually counted, and the water content was determined according to the formula: WC (%) = (FW − DW)/FW × 100. Here, FW is the fresh weight and DW is the dry weight [78]. Each biological replicate contained 40 plants of each genotype and the experiment was conducted in 3 replicates.

### 4.11. Plant Tolerance to Drought Stress

Two-week-old seedlings of 35S::ERF1 L1 and L2 lines and of the WT control were transferred to individual pots containing a vermiculite and humus (1:2) soil mixture and grown in a phytotron. Plants were watered with one-fifth-strength Hoagland solution, and they were weighed daily during the experiment. Well-watered control plants were grown at 100% field capacity (0% of water loss). The time course for the drought stress assay started by withholding the nutrient solution until reaching 70% water loss. The following analyses were performed for transgenic lines and WT tobacco plants: photosynthetic parameters, chlorophyll index, proline content, relative water content and quantification of the plant hormones abscisic acid, 6-dymethylamino purine, gibberellic acid- 1, jasmonic acid, and salicylic acid. Each biological replicate consisted of 20 plants of each genotype.

#### 4.11.1. Determination of Chlorophyll Index and Photosynthetic Analyzes

Measurements of foliar chlorophyll index were carried out with a SPAD-502 chlorophyll meter (Konica Minolta, Inc., Tokyo, Japan) on the second fully expanded leaf from plants of each genotype. In addition, analyses were also performed for net CO_2_ assimilation (photosynthesis) rate (A, μmol CO_2_ m^−2^ s^−1^), stomatal conductance to water vapor (Gs, mmol m^−2^ s^−1^), and transpiration (E, g/m^−2^ h^−1^) on the third pair of fully expanded leaves below the apex, with four biological replicates. Leaf gas exchange were measurements from 8:30 a.m. to 12:00 p.m., at 25 °C, with a portable infrared CO_2_/H_2_O gas analyzer (IRGA) (LCpro-32 070; ADC Bioscientific Ltd., Great Amwell, U.K.) equipped with a broad leaf chamber. The Photosynthetically Active Radiation (P.A.R.) was adjusted to 1000 μmol m^−2^ s^−1^ at 25 °C. Physiological parameters were registered in transgenic plants and WT plants under water deficit and well-watered conditions.

#### 4.11.2. Measurement of Chlorophyll Fluorescence

Chlorophyll fluorescence was measured with a chlorophyll fluorimeter (IMAGIM-PAM M-series; Heinz Walz, Effeltrich, Germany). The minimum and maximal fluorescence yield of the tobacco leaves were monitored in dark-adapted leaves (20 min). Kinetic analyses were carried out with an actinic light (81 µmol quanta m^−2^ s^−1^ PAR), and repeated pulses of saturating light at 2700 µmol quanta m^−2^ s^−1^ PAR for 0.8 s, and at intervals of 20 s [79]. The following parameters were also analyzed: the actual photosynthetic efficiency (Y(II)); quantum yields of non-regulated energy dissipation Y(NO); the quantum yield of regulated energy dissipation (Y(NPQ)); and the coefficient of actinic light quenching (qL) [80]. We measured all the chlorophyll traits using the second fully expanded leaf from the apical meristem of transgenic plants and WT plants under water deficit and well-watered conditions.

#### 4.11.3. Proline Content and Relative Water Content Measurements

The proline content was measured by using a modified method described by [81]. Approximately 100 mg of frozen leaf samples were incubated overnight immersed in 10 mL of 3% (w/v) sulphosalicylic acid, at 4 °C on a shaker, and a 2.0 mL aliquot was used for the acid ninhydrin reaction. The absorbance was read at 520 nm against a toluene blank on UV–visible spectrophotometer (Amersham Bioscience; Ultrospec 2100 pro). Proline concentration was determined by using a calibration curve. The relative water content (RWC) was evaluated according to the formula: RWC (%) = (FW−DW)/(TW−DW) × 100, where FW is the fresh weight, TW is the abbreviation for means of turgid weight, and DW is the dry weight [82]. For all analyzed parameters, we used the third fully expanded leaf from the apical meristem of transgenic plants and WT plants under normal and water deficit conditions.

#### 4.11.4. Plant Hormone Quantification

The second leaf of each plant under water deficit and well-watered conditions was harvested at the treatment conclusion. Samples were flash-frozen using liquid nitrogen and kept at −80 °C. Extracts of plant hormones were obtained as described by [83]. Ten microliters of each extract sample were injected into a UHPLC–MS system consisting of an Accela Series U-HPLC (ThermoFisher Scientific, Waltham, MA, USA) equipped with an Exactive mass spectrometer (ThermoFisher Scientific, Waltham, MA, USA) that uses a heated electrospray ionization (HESI) interface. Mass spectra were obtained using the Xcalibur software version 2.2 (ThermoFisher Scientific, Waltham, MA, USA). For quantification of the plant hormones (abscisic acid, 6-dymethylamino purine, gibberellic acid-1, jasmonic acid, and salicylic acid), calibration curves were constructed for each analyzed hormone (1, 10, 50, and 100 μg L^−1^) and corrected for 10 μg L^−1^ deuterated internal standards. Recovery percentages ranged from 92% to 95%.

### 4.12. Expression Analysis of TgERF1-Transgenic Plants under Drought Stress

For the expression analysis of transgenic plants under drought stress, two-week-old seedlings of WT and 3 lines 35S::ERF1 were transferred individually to trays containing soil mixture (vermiculite: humus = 1:2). Then, four-week-old plants were subjected to severe drought stress by withholding water for 10 days’ drought conditions. Total RNA was isolated from leaf tissues of 35S::TgERF1 lines and WT under 10-d drought conditions and cDNA synthesis was performed as described above (4.4). The transgene expression was analyzed through RT-qPCR on an ABI 7500 qPCR thermocycler (Applied Biosystems, USA) using the Platinum SYBR Green Supermix (Invitrogen, USA). Cycling conditions consisted of 95 °C for 10 min, followed by 40 amplification cycles of 95 °C for 15 s, 60 °C for 30 s, and 72 °C for 30 s. *TgERF1* was the target gene and RT-PCR used the TgERF1RT_F and TgERF1RT_R primers (Table S3). The constitutive Elongation Factor-1 alpha (NtEF-1 alpha) housekeeping gene was an internal control using the primers NtER-1AF and NtEF-1AR (Table S5). The comparative ΔΔCt method was used to calculate relative expression levels in Real-Time qPCR data. Changes in gene expression under stress treatments were described in previous studies, such as genes involved in antioxidant-related response (NtAPX, NtCAT, and NtSOD) [84] and genes combating cellular dehydration (NtLEA5, NtERD10C) [68,84,85]. In the present work, these genes were also characterized in studied tobacco plants by RT-qPCR using primers listed in Table S6. Each experiment was carried out with three biological replicates and two technical replicates. Each biological replicate consisted of 4 plants of each genotype grown in a phytotron.

### 4.13. Statistical Analysis

Transformation experiments were performed in a completely randomized design with at least three independent replicates. Data from transgenic lines overexpressing the *TgERF1* gene WT plants were analyzed with ANOVA and Dunnett’s tests using GraphPad Prism 5 software (La Jolla, CA, USA). Statistical significance was set at *p* < 0.05. Data are reported as means ± Standard Error of the Mean (SEM).

## 5. Conclusions

The *TgERF1* gene, which encodes an AP2/ERF transcription factor, was successfully isolated from *T. grandis*. *TgERF1* and the transgenic lines were subjected to osmotic and saline abiotic stresses and phytohormone treatments (PEG, NaCl, ABA, ACC, and MeJA). The *TgERF1* expression was exclusive to the nucleus, which is in accordance with the fact that this gene does not present transmembrane motifs and with its role as a transcription factor. Overexpression of *TgERF1* in tobacco enhanced tolerance to salt and drought stresses. The improved stress tolerance of transgenic plants compared to control plants was supported by morphological and physio-biochemical data, and by activation of an array of stress-responsive genes, analyzed at molecular level, leading to the synthesis of protective compounds. Results presented here demonstrate that the *TgERF1* overexpression significantly altered drought stress tolerance in transgenic plants and increased our understanding of the role played by the TgERF1 transcription factor in responses to abiotic stresses. All the evidence presented here indicates that *TgERF1* is a promising candidate gene to be used as a selective marker on plant breeding aiming to improve plant stress tolerance.

## Data Availability

Not applicable.

## Supplementary Materials

The following supporting information can be downloaded at: https://www.mdpi.com/article/10.3390/ijms24044149/s1.
