# Supplementary material for: Overexpression of TgERF1, a Transcription Factor from Tectona grandis, Increases Tolerance to Drought and Salt Stress in Tobacco
Source: Int J Mol Sci. 2023 Feb 19;24(4):4149. doi: 10.3390/ijms24044149 (PMC9961280; doi:10.3390/ijms24044149)
Supplement: Supplementary file 1 [file ijms-24-04149-s001.zip › ijms-2202096-supplementary.pdf]

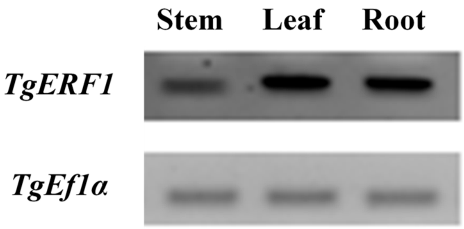

Figure S1. Detection of the *TgERF1* transcript in diverse tissues of teak plants. Tissues samples were examined using a semiquantitative RT-PCR characterization. The constitutive *TgEF1α* (*Elongation Factor-1 alpha*) housekeeping gene was used as an internal control. TgERF1, a ERF transcription factors from *Tectona grandis* confers Salt and Osmotic Stress tolerance to *TgERF1* transgenic tobacco.

Table S1. Prediction of the subcellular localization of TgERF1 by WOLF.

ID	Site	Lenght
TgERF1	Nucl.	166
At2g22310.1	Nucl.	365
At5g18450.1	Nucl.	307
At4g39910.1	Nucl.	371
At2g06330.1	Nucl.	555
ORC2_ARATH	Nucl.	363
At5g67010.1	Nucl.	162
At5g63870.1	Nucl.	413
At1g23240.1	Extr.	184
ILLA_LEUGL	Extr.	137
At5g50470.1	Nucl.	212
At3g18485.1	Cyto.	386
At4g39780.1	Nucl.	272
At1g12360.1	Cyto.	666
SBP2_ANTMA	Nucl.	171

Table S2. Transmembrane prediction the TgERF1 protein through the HMMTOP Protein: TgERF1.

Length: 171
N-terminus: OUT
Number of transmembrane helices: 0
Transmembrane helices: 0

Total entropy of the model: 17.0129
Entropy of the best path: 17.0129

The best path:

```
seq   LCLRFMKQMK NLTKEEFVHI LRRQSTGFSR GSSKYRGVTL HKCGRWEARM     50
pred  OOOOOOOOOO OOOOOOOOOO OOOOOOOOOO OOOOOOOOOO OOOOOOOOOO

seq   GQFLGKKAYD KAAIKCNGRE AVTNFEPSNY DREINISSRD GGSGSGLDLN    100
pred  OOOOOOOOOO OOOOOOOOOO OOOOOOOOOO OOOOOOOOOO OOOOOOOOOO
```

```
seq   LGISLSSDGP QGNDTTRNLH FSHPSGELPD GKRLKVLLSL HKMLKLATVF    150
pred  OOOOOOOOOO OOOOOOOOOO OOOOOOOOOO OOOOOOOOOO OOOOOOOOOO

seq   QLSPFCSSCT MKFILNFQQA D   171
pred  OOOOOOOOOO OOOOOOOOOO O
```

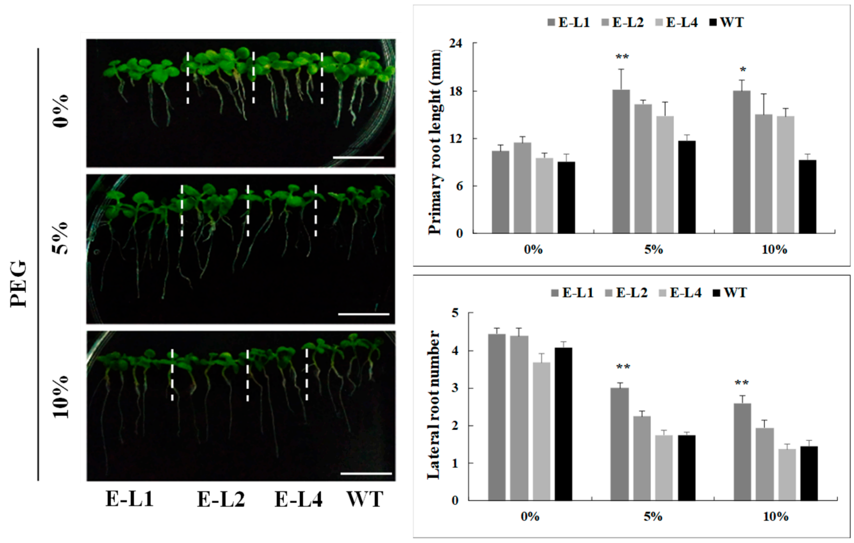

Figure S2. Root growth in seedlings of transgenic tobacco overexpressing the *TgERF1* gene and the wild type (WT) control under PEG stress. Three-day-old seedlings were transferred to 0,5% and 10% PEG treatments and grown vertically for seven days. The primary root length and lateral root numbers were recorded at seven days after the transfer. Bar: 1 cm. For each-experiment 48 seedlings/line were used. Bars represent the mean value ± SEM of three independent assays. Significant differences were determined by ANOVA followed by Dunnett's test at $p < 0.05$ and $p < 0.01$.

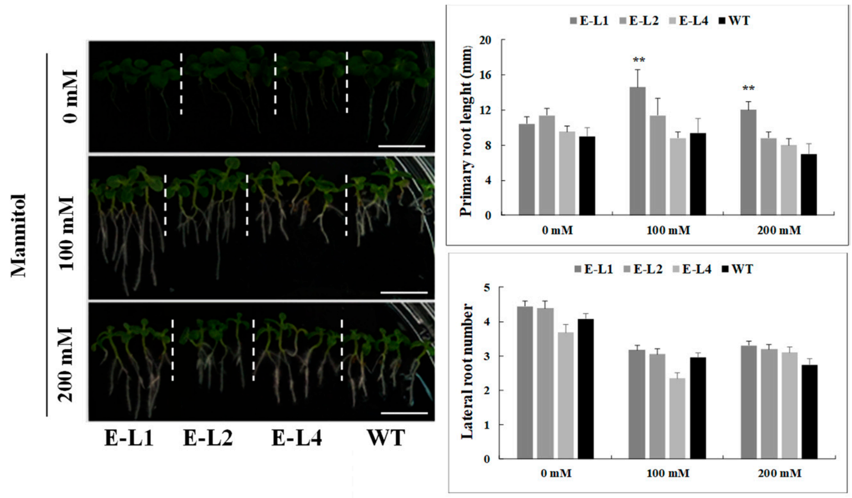

Figure S3. Root growth in seedlings of transgenic tobacco overexpressing the *TgERF1* gene and the wild type (WT) control under mannitol stress. Three-day-old seedlings were transferred to 0, 100 and 200 mM mannitol treatments and grown vertically for seven days. The primary root length and lateral root numbers were recorded at seven days after the transfer. Bar: 1 cm. For each-experiment 48 seedlings/line were used. Bars represent the mean value ± SEM of three independent assays. Significant differences were determined by ANOVA followed by Dunnett's test at $p < 0.05$ and $p < 0.01$.

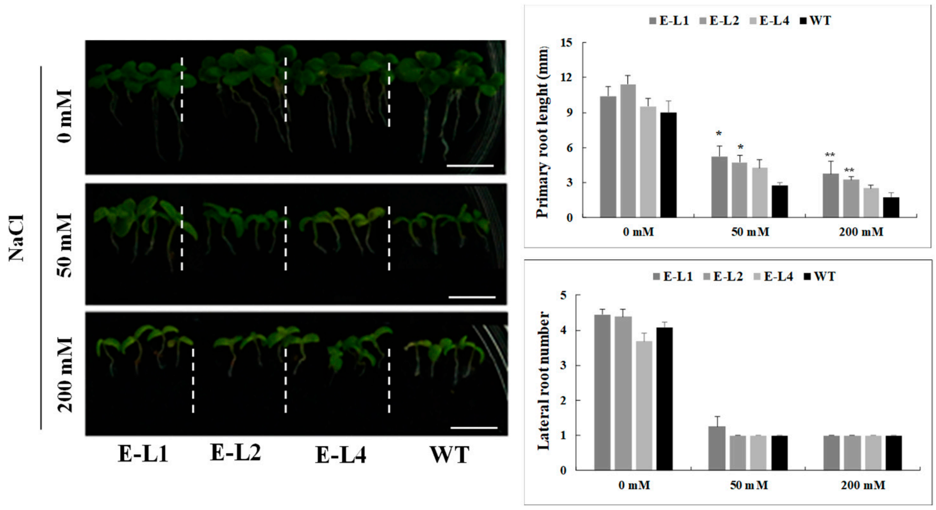

Figure S4. Root growth in seedlings of transgenic tobacco overexpressing the *TgERF1* gene and the wild type (WT) control under NaCl stress. Three-day-old seedlings were transferred to 0, 50 and 200 mM NaCl treatments and grown vertically for seven days. The primary root length and lateral root numbers were recorded at seven days after the transfer. Bar: 1 cm. For each-experiment 48 seedlings/line were used. Bars represent the mean value ± SEM of three independent assays. Significant differences were determined by ANOVA followed by Dunnett's test at $p < 0.05$ and $p < 0.01$.

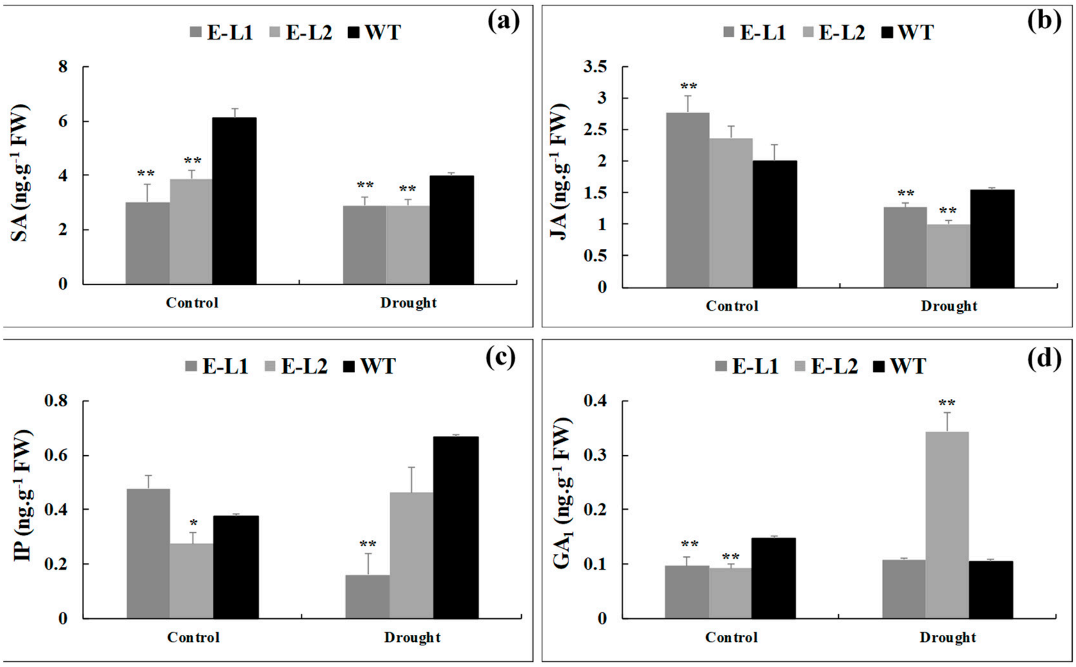

Figure S5. Endogenous hormones levels in leaves of *TgERF1* transgenic tobacco and WT control in response to drought stress. Plants were grown in individual pots, watered with one-fifth-strength Hoagland solution and weighed daily. Well-watered control plants were grown in 100% field capacity (0% water loss). The time course drought stress assay started by withholding the nutrient solution until reaching 70% water loss. **(a)** Salicylic acid; **(b)** jasmonic acid; **(c)** 6-dymethylamino purine – iP; **(d)** gibberellic acid-1. Leaf samples were obtained from plants under well-watered and water-deficit conditions, Primary root length and lateral root numbers were recorded. In each experiment 16 seedlings/line were used. Bars represent mean value ± SEM. Significant differences were determined by ANOVA followed by Dunnett's test at $p < 0.05$ and $p < 0.01$.

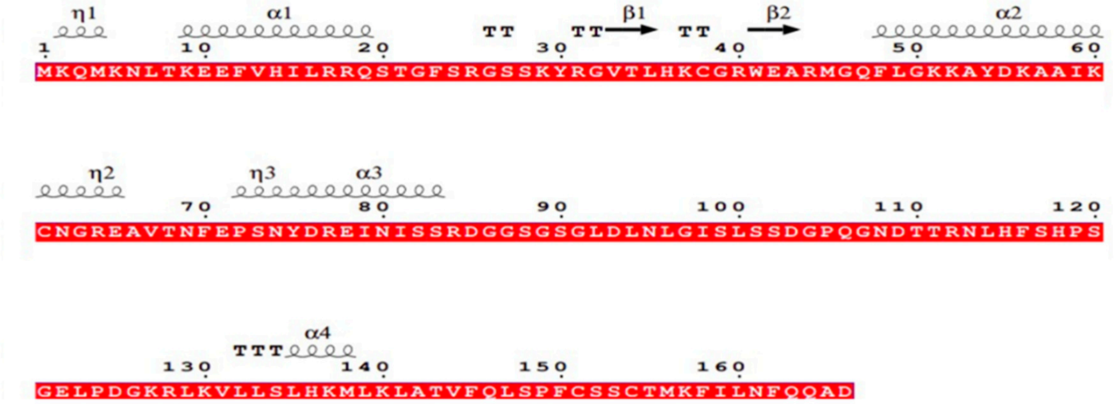

Figure S6. TgERF1 Protein sequence and secondary structure. n = loop, α = Alpha-Helix and β = Beta-Pleated sheets. The secondary prediction was used ESPrint 3.0

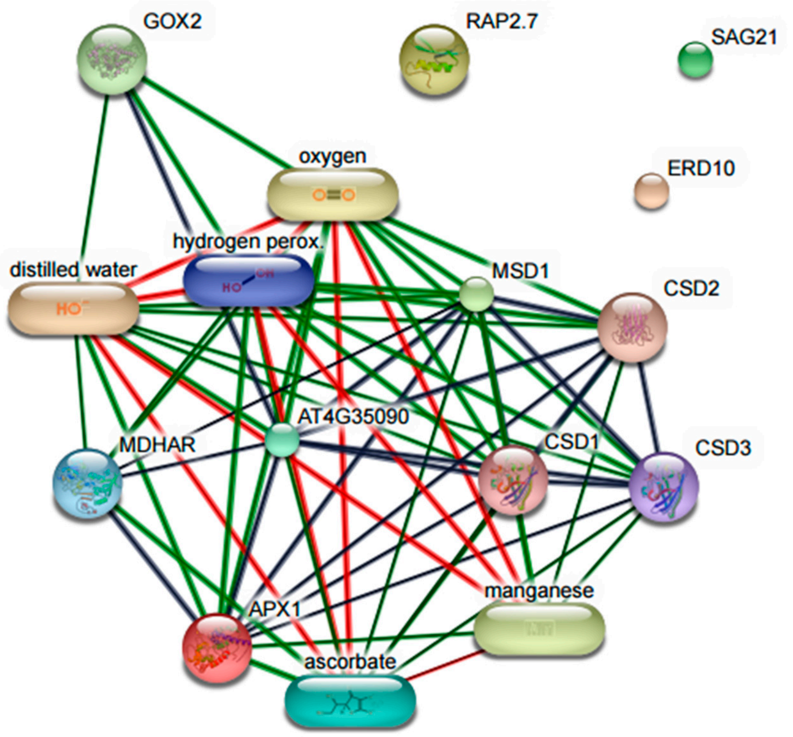

Figure S7. Analysis of *TgERF1* gene network generated in Cytoscape and showing association among significantly modulated genes of Figure 13. Arabidopsis orthologous genes were used. Genes showing no association are placed separately from the gene network. Different colored edges with arrows show direction of interactions. *TgERF1* - AT2G28550-RAP2.7 - related to AP2.7, *NtAPX* – *APX1* AT1G07890.8, *NtSOD1* – MSD1 AT3G10920.1, *NtCAT* - AT4G35090 - catalase 2, *NtLEA5* – AT1G20450.1 - *ERD10* - dehydrin ERD10, *NtERD10C* - AT4G02380.1' *SAG21* - senescence-associated gene 21.

Table S3. Primers used in PCR and RT-qPCR analysis of Tg*ERF1* in *Tectona grandis* under stress

Gene Name	Accession Number	Primer	Sequence (5'-3')	Amplicon Size (bp)	Reference
TgERF1	MH003850.1	TgERF1RTF	GGAAGAGAAGCCGTCACGAA		
		TgERF1RTR	ATCAAGACCGCTGCCACTAC	93	This work
**TgEF1 alpha**		TgEF-1AF	CATCAACATTGTGGTCATTGG		
		TgEF-1AR	CCAGACGCCTGTCAATCTTG		[30]

Table S4. Primers used to amplify the cDNA of *TgERF1* from *Tectona grandis*

Gene Name	Accession Number	Primer	Sequence (5'-3')	Amplicon Size (bp)	Reference
TgERF1	MH003850.1	TgERF1CDSF	CACCATGAAACAGATGAAGAATTTAACA	498	This work
		TgERF1CDSR	GTCTGCTTGCTGAAAATTAAGAATGAAT		

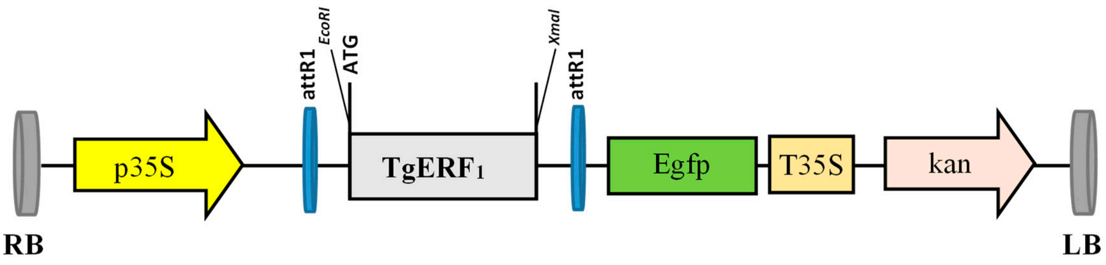

Figure S8. Vector used for transformation of tobacco plants. To construct a TgERF1- GFP fusion protein, the *TgERF1* coding region without the stop codon was inserted in frame into *EcoRI* and *XmaI* sites of pK7FWG2 upstream *EGFP* coding region. Driven by CAMV35S promoter (p35S), transcription of the fusion gene of *TgERF1* and GFP will be expressed after the construct was transformed into tobacco leaves. The pK7FWG2 contains the selective marker *neomycin phosphotransferase II* (*nptII*) that gives resistance to kanamycin.

Table S5 Primers used in PCR and RT-qPCR analysis of Tg*ERF1* overexpression transgenic tobacco (*Nicotiana tabacum*).

Gene Name	Accession Number	Primer	Sequence (5'-3')	Amplicon Size (bp)	Reference
35S	-	P35SPCRF	CGCACAATCCCACTATCCTTC		
				520	This work
TgERF1	MH003850.1	TgERF1RTF	GGAAGAGAAGCCGTCACGAA		
		TgERF1RTR	ATCAAGACCGCTGCCACTAC	93	This work
NtEF1 alpha	AF120093	EF-1AF	TGAGATGCACCACGAAGCTC		[77]
		EF1-AR	CCAACATTGTCACCAGGAAGTG		

Table S6. Primers used to analyze expression levels of stress responsive genes of *TgERF1* lines

Gene Name	Accession Number	Primer	Sequence (5'-3')	Reference
NtAPX	U15933.1	NtAPXF	GTTTGGGCTTTTCTCCTCGAC	[84]
		NtAPXR	GGAGCATAAGAGGAGCGCAA	

NtCAT1	U93244.1	NtCAT1F<br>NtCAT1R	GGCCGCTACAACTCTCTCTTT<br>ACAGGACCTCTTGCACCAAC	[84]
**NtSOD**	AB093097.1	NtSODF<br>NtSODR	TCCCCTACGACTATGGAGCA<br>CGGTATGCAATTTGGCGACG	[84]
NtERD10C	AB049337.1	NtERD10CF<br>NtERD10CR	GGATTGTCTCCTGCTGCTGT<br>GCTCTTCTAATAACTCAGCACCC	[84]
NtLEA5	AF053076.1	LEAF<br>LEAR	TTGTTAGCAGGCGTGGGTAT<br>CTCTCGCTCTTGTTG GGTTC	[85]

Table S7. Full length of the *TgEREF1* gene sequence related with drought stress. *TgERF1* corresponds to name “comp21250_c1_seq6” in the root transcriptome.

comp21250_c1_seq6 (2548 pb)
>*TgEREF1*
GGGAATCCCATATCTGGTTCGTCTGTACATACATATACATACATATGGACTAATCTGAATATAG
TCTTCTGTATATTGCATGCATGTATGTGTTGTCTGTAGATTGTGCTTGCTATGGTTAGACAACCTC
TTTCCTTACATTATGCTGCGATATTTTAATTTCCGCAGTCAACAAATGATTGTTATTTTCCAAAGT
TTTTCTCAAAATTAGCAGCCTAGTATCAAGAAACTTGGAAATATTGTTAGTATTTCTTGTGGATG
TGTTGCTCTATTGGGATTTGTCTGTGGAAGCATGAGTGTTAAATCTGTTCATGGGCTACTTATTTA
TTGTTATATGCTCCTTGATAAAATCTGTTCATGGGCTCTACTTATTTATTGTTATATGCTGCTTGAT
TAGGTTAATTTGGGTGGCAATTTGTGTACGGTGATCGTCTGAATTTGTGATTTTAGCTGAATCTTG
GCGTTTTCTTGCAGGGATTGTGGCAAACAAGTCTATTTGGGAGGATTTGACACAGCTCATGCTG
CAGCTAGGGCATATGATCGTGCTGCAATCAAGTTTCGGGGAGTTGATGCTGATATCAATTTCAA
TATCAGTGATTATGAGGAGGACATGAAACAGATGAAGAATTTAACAAAAGAAGAGTTTGTTCA
CATTCTTCGCCGCCAAAGCACTGGATTCTCTCGGGGGAGCTCAAAGTACAGAGGAGTAACCCT
GCACAAATGTGGTCGTTGGGAAGCACGAATGGGCCAATTCCTCGGAAAGAAGGCTTATGATAA
AGCAGCCATTAAATGCAACGGAAGAGAAGCCGTCACGAACTTTGAGCCTAGTAACTATGACAG
GGAGATAAATATCAGTTCCAGAGACGGAGGTAGTGGCAGCGGTCTTGATCTCAACCTTGGAAT
TTCTTTATCTTCAGATGGTCCACAGGGGAATGACACTACCAGGAATTTGCACTTTTCTCATCCGT
CTGGCGAATTGCCTGATGGGAAGAGATTGAAGGTACTCTTATCCCTTCATAAAATGCTTAAATT
GGCTACAGTTTTTCAACTCTCCCCTTTTTGTTCCTCTTGTACTATGAAATTCATTCTTAATTTTCAG
CAAGCAGACTAGAGGGCTTCACTCCATTGATATATAATTGTTACTGCTGGCATAGAAGCAAATG
CTACATAAATTTTCTTTTCCTCCTTTTCTCTTCTTTTACACATATCTCAAGTGGTATAGATGCTATT
TGTTGGTCTCTCTCTTCTGAGAGTATTGAGCTAGGTAGTGCATCCAGACTCATGAAAACTGATAC
ACAATTTCATAGGTCGAACCGACCTCTGCTTCTCCTCAAGAAATCGTAATGTCAACCAAGTGTC
CTCCAATGTGGGCTGGCATATATTCTGGTTTTGCCCCAAATAGTAAGGTGATTGTTCTTTTCACA
TTTCTATTCAAAACCTTATTACTGCAGCCAAGTTGAGGTCTTGTTAGTACTGTCTTTAGACTTGCA
ACTTTCCCAAGTTACATGTTTCCTTACCAAAATTGATCATCCCCAGTACCATCTGTATTGTTTACT
TGTGGCTTGAATTGCCACCCATTCTTGCTCTCTCGTGTCAGTTATGAATAGAATGGTGCAATGAC
CTTATGAGAGTCACCTGTGAACTCTTTACATAGTTTCAGTTTCTTTGTTCGTTTTAAGTGTTGTCA
TCTGCAACCAACTATTGAGTTAGAAGAAAGTCCTTTGCACTTTTTGCTCTTGTTTTTCCACTTCGT
ATTTTTCCTGCTTCAGGATGGGGGTGGGAGAAATAGAATTTGATATCAGCAGTTTTCTTGACAC
ATTCATTGAAGGAGAGAAGCAGATATATCAGAAGAAATGCCAAGTCTGTAATAAAAGATGTGT
CATTAAGTTTGCAGTTATGGATAACAATTTAATTTTGACTGACAGGAAATAGCAACGAGAATGG
GCGCTGAAGCTGTTTCCCTGCCTGGATTTTCAAACTGGCCATGGAAAATGCCAAGCCATGGTGC
AGTCACCCCTGTGCCACTTTTGTCTTCTTCTGCAGCATCATCAGGATTCTCTTCCGCGACCTCTCC
ATACTTGAATCCCCTCATGTCATTGACATCTAGAGCGCAATTTCCGGCAACTGCTTTCCGCAGCC
TTCAGTGAAACTTAGCTCCAACAGGAGCAACTGAAACAACATCAGCATTGGGAAAAGCCGCA
ATTTATTCTGTTCAAGATTTGGCTATTAAGAAAAGCCACGCCAATCAGAGATTCATCTCCTCAG
CAATACATATAATACGAATAACAGATTGACTCAGTCATATGATGTCTCGATTGGTACAATGCTC
ATTAGAATTTTGGAGTCTCAATATTGTGTAGATTCTGATTTGGCAAAGTAAATGGATTGCTGTTC
ATAATCTGTCGTGAATTCCAGTGTGAATCTTTAGCTAGATAAGAAATGCAACAAAAAGAAGTA
AAACCGTGGAATCTCGGGTACGCTTTAGGTGCCGGGCACATAAATTTGCGTGATTTGAGAAGCA
TTTGACCTTTCGTTTACATATCAATTT
